# Unprecedented expansion of graphite with low power laser for high-quality liquid phase exfoliated graphene

**DOI:** 10.1038/s41598-025-17947-6

**Published:** 2025-09-24

**Authors:** Rami Elkaffas, Baosong Li, Shanavas Shajahan, Basel Altawil, Zeyad M. Abdulhamid, Dalaver Anjum, Yarjan Abdul Samad

**Affiliations:** 1 Department of Aerospace Engineering, Khalifa University of Science and Technology, 127788 Abu Dhabi, United Arab Emirates; 2 Department of Physics, Khalifa University of Science and Technology, 127788 Abu Dhabi, United Arab Emirates; 3 Cambridge Graphene Center, University of Cambridge, Cambridge, CB3 0FA UK

**Keywords:** Expanded graphite, Graphene, 2D materials, Electromagnetic interference shielding, Engineering, Materials science

## Abstract

As graphene and related materials increasingly integrate into various industries, producing high-quality graphene sheets on a large scale becomes crucial. Here, we present a unique approach to the large-scale production of graphene through laser-assisted graphite expansion followed by ultrasonic exfoliation. The present method utilizes laser technology to significantly expand graphite, achieving an expansion rate of 800 mL g^−1^ with a deficient energy consumption of just two watts laser. The graphene produced via ultrasonication of expanded graphite samples exhibited high quality (I_D_/I_G_) ~ 0.13 and a few layers with a (I_2D_/I_G_) ~ 0.52. Free-standing films with different thicknesses (11–69 µm) were successfully prepared through filtration, reaching significant electrical conductivity up to (~ 1707 S cm^−1^). The as-prepared graphene films are further explored for electromagnetic interference (EMI) shielding. The highest shielding effectiveness (SE) was recorded for the graphene film with a thickness of 69 µm, reaching a value of ~ 72 dB. Meanwhile, the graphene film with an 11 µm thickness achieves the highest absolute effectiveness (SSE/t) of ~ 58,666 dB cm^2^ g^−1^_,_ surpassing most current graphene and MXene films, which typically present values in the range of 10,000 to 40,000 dB cm^2^ g^−1^. This present laser-assisted expansion incorporating a sonication exfoliation strategy paves a new way to produce graphene on a large scale.

**Supplementary Information:**

The online version contains supplementary material available at 10.1038/s41598-025-17947-6.

## Introduction

Graphene, a single-layer carbon allotrope arranged in a hexagonal lattice, has garnered significant attention from academia ^1^ and industry ^2^ in recent years. The isolation of graphene by Novoselov et al. ^3^ through micromechanical cleavage of graphite with scotch tape marked a pivotal moment. Their groundbreaking work led to the Nobel Prize in 2010. Graphene’s 2D hexagonal carbon lattice structure gives its distinct properties, including a remarkable tensile strength of 130 GPa and a Young’s modulus of 1.0 TPa ^4^, exceptional thermal conductivity at 5 × 10^3^ W mK^−1^ ^5^, electrical conductivity up to 80 MS m^−1^ ^6^, a high surface area of 2630 m^2^ g^−1^ ^7^, outstanding electron charge transport properties (~ 200,000 cm^2^ V^−1^ s^−1^) ^8^, and excellent optical characteristics with an absorption rate of about 2.3% over a wide wavelength range ^9^. These exceptional properties have made graphene a potent candidate for a variety of applications, including electronic devices ^10^, optical devices ^11^, electrochemical sensing ^12^, biosensing ^13^, energy conversion ^14^, energy storage, including supercapacitors and batteries ^15,16^, water treatment ^17^, desalination ^18^, medicine ^19^, military ^20^, coating ^21^, printing ^22^, catalysis ^23^, photocatalysis ^24^, advanced composite material ^25^. There are two main types of graphene preparation methods: bottom-up and top-down. The bottom-up approach is growing graphene continuously by breaking the chemical bonds of carbon-containing compounds and depositing carbon atoms on the substrate by certain methods, such as chemical vapor deposition ^26^, epitaxial growth ^27^, and plasma synthesis ^28^. Top-down methods obtain graphene from bulk synthetic or natural graphite by breaking the van der Waals forces between graphite layers with the assistance of external forces ^29^.

The top-down methods mainly include exfoliation via ultrasonic waves ^30^, ball milling ^31^, high shear delamination ^32^, high-pressure homogenization ^33^, microfluidics ^34^, and electrochemical intercalation ^35^. The intercalation-assisted exfoliation of graphite to graphene offers several significant advantages, such as the versatility of the method by using different intercalants, including anions such as SO_4_^2−^ ^36^, BF_4_^−^^37^, ClO_4_^−^^38^ and C_2_O_4_^2−^ ^39^ and alkali earth metals such as Li ^40^, K ^41^, and Na ^42^, the properties of graphene can be tailored, offering the possibility to modify its electronic structure ^43^ or enhance its reactivity ^44^. Additionally, it enables the scalable production of graphene ^45^, making it an attractive method for large-scale applications ^46^.

**Fig. 1:**
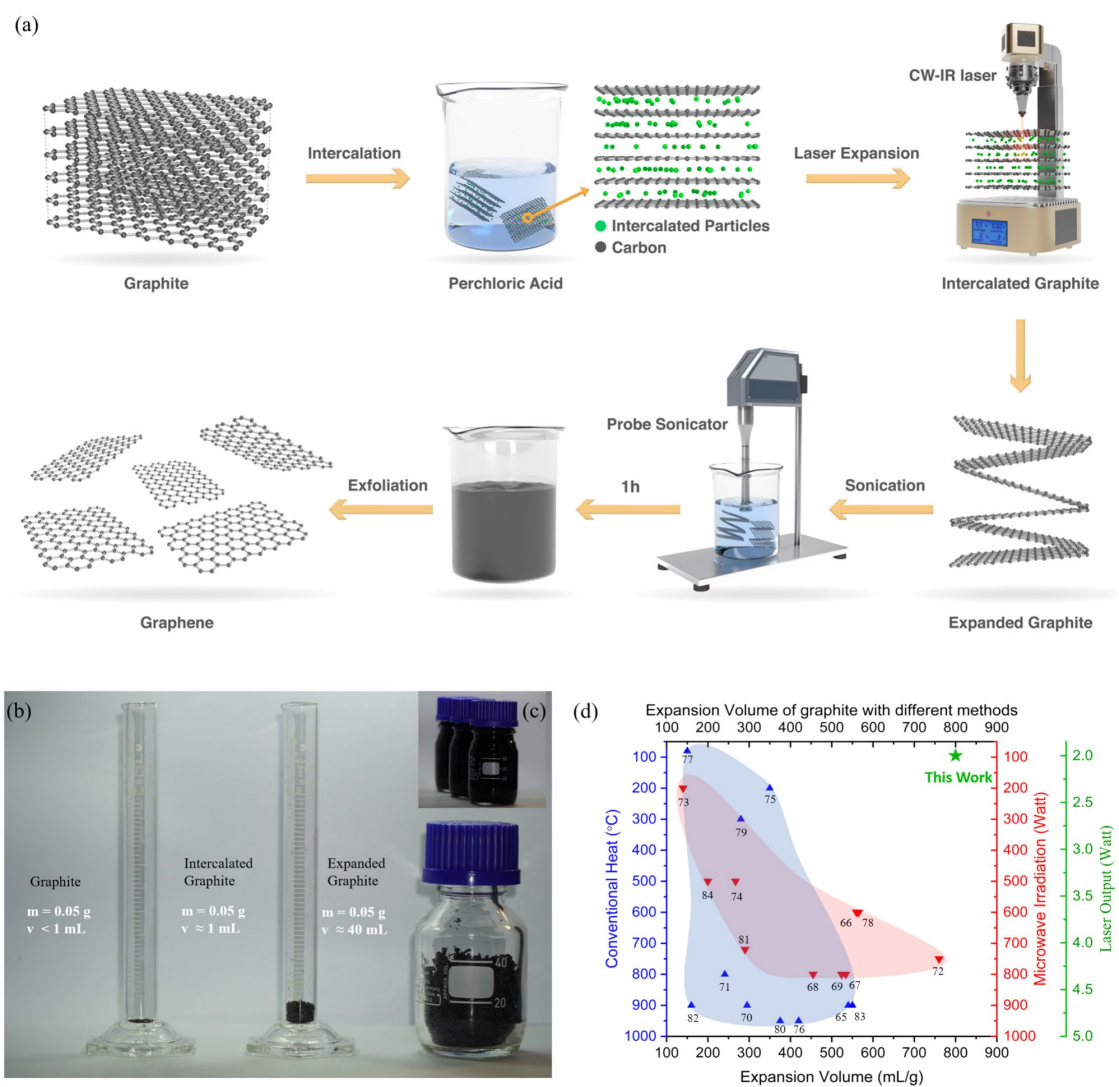
(**a**) Schematic illustration of the preparation of the laser-assisted exfoliated graphene. (**b**) Mass and volume of graphite, intercalated graphite, and expanded graphite. (**c**) Expansion volume of different methods from the literature ^47,65–83^.

The typical procedure of intercalation-assisted exfoliation involves the intercalation of a guest (such as ClO_4_^-^^47^ or SO_4_^−^^48^) and the subsequent exfoliation of the host (graphite). Guest intercalation is achieved via chemical routes, which form the graphite intercalated compound (GIC), while host exfoliation refers to isolating atomic layers from the intercalation compound (host + guest) ^49^. This exfoliation process begins with thermal treatment of the GIC to yield thermally expanded graphite (TEG), followed by sonication or any similar form of external forces to exfoliate TEG, which creates a suspension of the exfoliated nanosheets, allowing for their individualization and dispersion ^50^. Expanded graphite (EG) refers to graphite expanded along the c-axis up to several hundred times ^51^. EG can be produced through various methods utilizing different intercalation and oxidation agents. For instance, intercalation agents such as H_2_SO_4_^52^, HNO_3_^53^, or HClO_4_^54^ can be combined with oxidation agents like potassium permanganate ^55^, ammonium persulphate ^56^, or hydrogen peroxide ^57^ to facilitate the production process. The subsequent treatment can involve conventional heating ^58^, microwave heating ^59^, or room temperature processes ^60^ to achieve the desired EG structure. These methods come with several disadvantages. These processes necessitate substantial time consumption, particularly in the washing and drying stages. The intercalated graphite is washed for several hours to reach a neutral pH ^61^ and then oven-dried for approximately 12 to 24 h ^62^. The process of thermally expanding the intercalated graphite varies and requires temperatures ranging from 600 to 1200 degrees Celsius for heat expansion and power inputs between 350 to 800W for microwave expansion, as shown in Fig. 1c. To the best of our knowledge, this study is the first to achieve unprecedented intercalated graphite expansion using a laser source. This novel approach enhances the characteristics of the expanded graphite produced, significantly simplifying the exfoliation process and making it more efficient and scalable for various applications. In the present work, we report a unique approach for synthesizing graphene from natural graphite flakes under laser irradiation using perchloric acid intercalation, achieving an expansion volume of 800 mL g^−1^. Subsequently, the resultant expanded graphite is exfoliated into graphene sheets using probe sonication with a frequency of 24 kHz and power of 750 watts for 1 h. The graphene samples exhibited high quality (I_D_/I_G_) ~ 0.13 with few layers characteristic with a (I_2D_/I_G_) ~ 0.52. Free-standing graphene films with varying thicknesses from 11 µm to 69 µm have been fabricated and tested for their EMI shielding properties. They showed significant electrical conductivity reaching up to 1700 S cm^−1^ for the 11 µm thick film. SE for the films ranged between 20 dB for 11 µm thick film and 72 dB for 69 µm thick film. Compared to state-of-the-art graphene and MXenes that fall in the category of free-standing films, they showed significant absolute shielding effectiveness ranging from (~ 37,714 to ~ 58,666) dB cm^2^ g^−1^.

## Results and discussion

The expansion volume of expanded graphite is a crucial parameter that drives its exfoliation efficiency ^63^. The expansion process is pivotal for evaluating performance, influenced by variables such as the type of intercalant used, the degree of intercalation, and the expansion temperature ^64^. Figure 1c illustrates the expansion volume of graphite, measured in milliliters per gram (mL g^−1^) on the x-axis, and its correlation with three different metrics on the y-axis: conventional heat in degrees Celsius (°C), microwave irradiation in Watts (W), and laser output, also in Watts (W). It compiles results from previous literature, primarily focusing on perchloric acid as the intercalating agent. Conventional heating methods demonstrate an increase in expansion volume with temperature, starting slightly above 150 mL g^−1^ and just over 550 mL g^−1^ at the highest temperature recorded at 900 °C ^65^. In contrast, the microwave irradiation method demonstrates a relatively higher and more variable expansion volume, ranging from 150 to 750 mL g^−1^, which correlates proportionally to the increasing microwave power applied between 200 and 800W. This pattern suggests that microwave irradiation may be more effective than conventional heating for expanding graphite. The present research introduces an advanced laser technique to expand the intercalated graphite. This innovative method has achieved the highest expansion volume recorded for expanded graphite at 800 mL g^−1^ while utilizing the lowest amount of power (2 W) compared to other methods. These findings emphasize the superior efficacy of laser-induced expansion, highlighting its potential as the optimal technique for graphite expansion for subsequent exfoliation to graphene due to its unparalleled volume increase and energy efficiency. Figure 1b shows the expansion ratio of graphite into expanded graphite with the same mass calculated using Eq. (1).

**Fig. 2:**
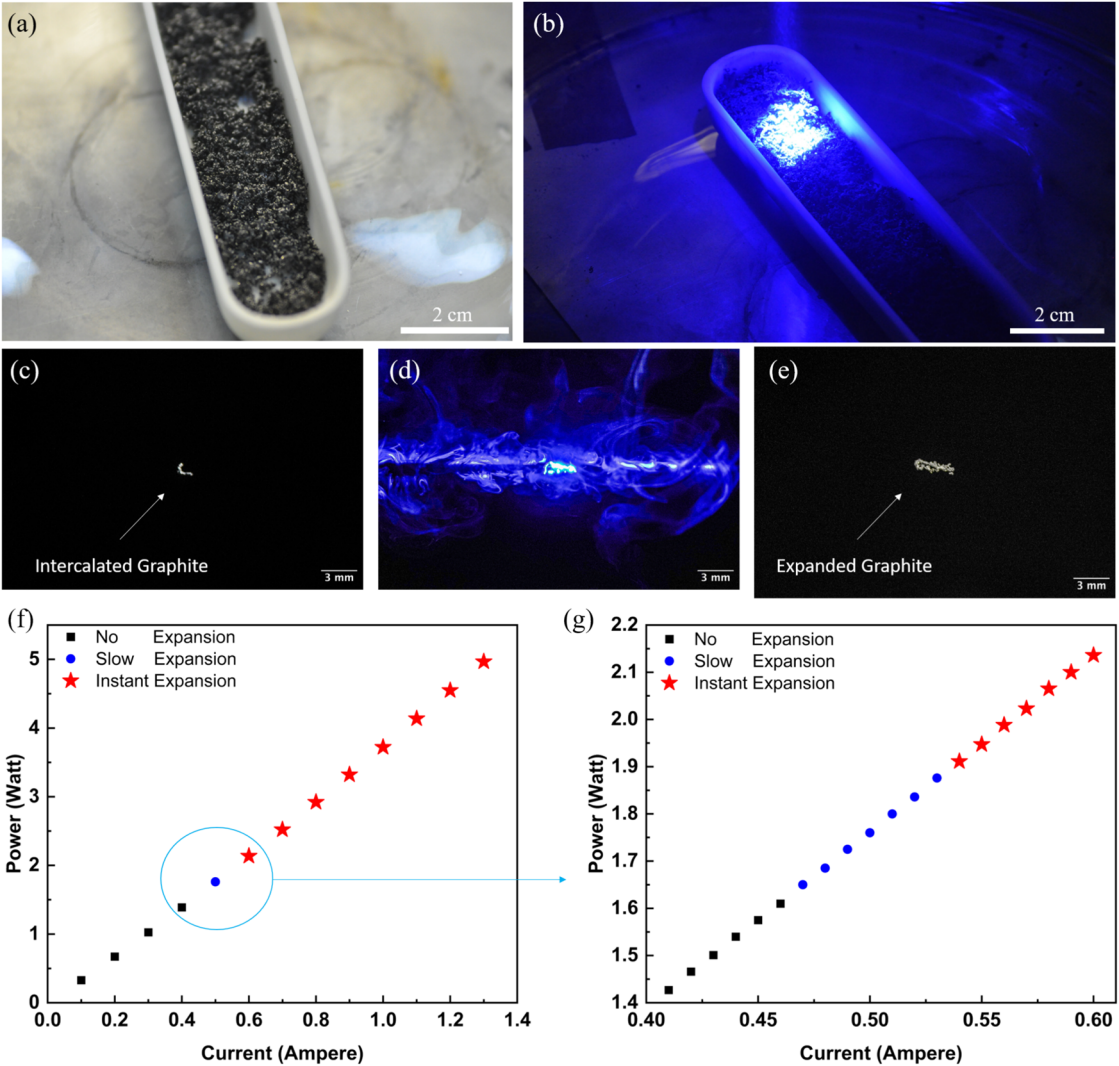
(**a**) Intercalated graphite before laser irradiation. (**b**) Intercalated graphite during laser irradiation. (**c**) A single flake of intercalated graphite before laser irradiation. (**d**) Flake is being irradiated with a laser and started to expand. (**e**) Expanded graphite flake after laser irradiation. (**f**) Laser optimization analysis as a function of Ampere vs Watt with a constant voltage of 3.5 Volt. (**g**) Detailed analysis of the optimization in a smaller range.

**Fig. 3:**
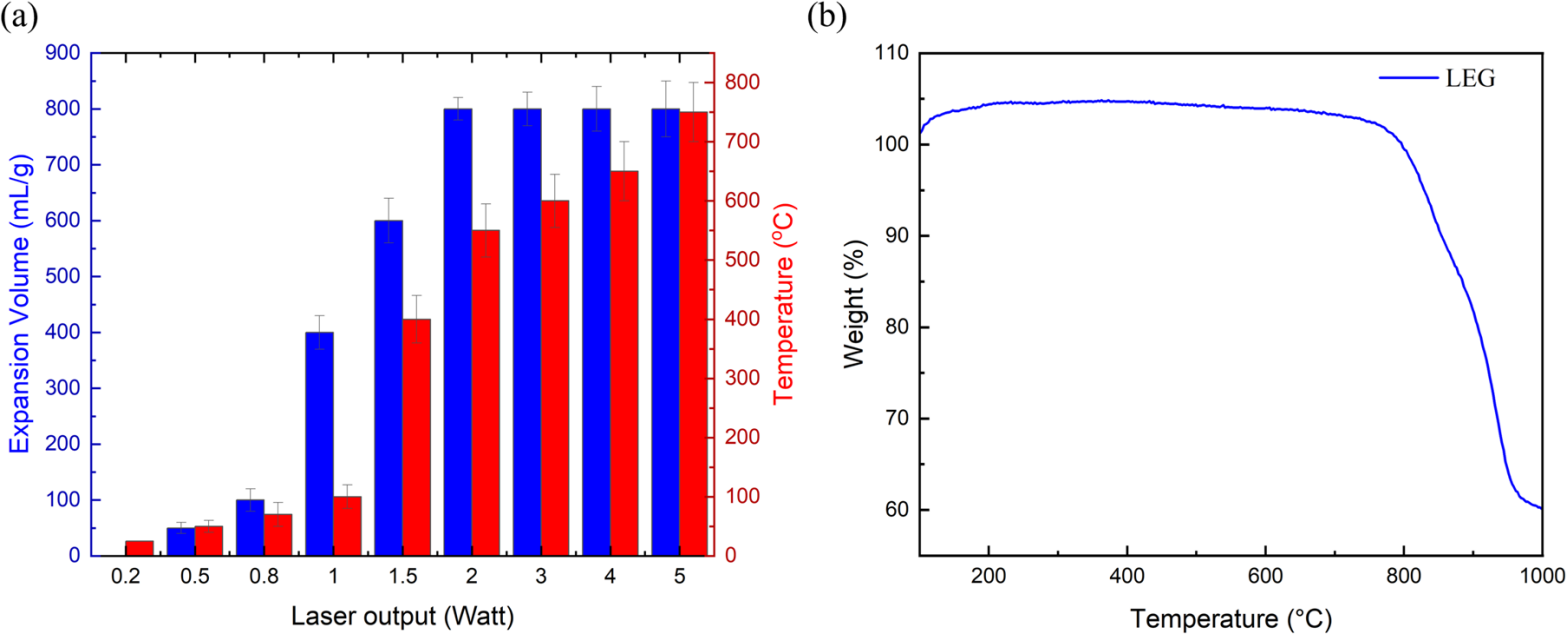
(**a**) Parametric study relating laser output in (watts) with Expansion volume in mL g^−1^ and the arising temperature from the interaction. (**b**) TGA of laser-expanded graphite.

The three methods—laser, microwave, and conventional heating—exhibit significant differences in expansion volume, cost, and energy efficiency. Comparative performance analysis of synthesis methods across six criteria was done in a diagram Figure S5, using ranges derived from literature values compiled in Tables S4 and S5. Figure S4 is a photograph of the laser setup. The continuous-wave blue laser (CW-blue laser) is directed at the intercalated graphite; when the laser is on, the localized heat from the laser heats the GIC to high temperatures, causing perchloric acid to vaporize from within the graphite layers, as shown in Fig. 1a. A suction fan swiftly removes these vapors, ensuring the operator’s safety and preserving the experiment’s integrity. The ceramic tube, being highly resistant to heat and not prone to damage from the laser, serves as a barrier to protect the surrounding area, including the work surface or floor, from accidental laser exposure. Surrounding the ceramic tube, a container collects the lightweight laser-expanded graphite produced. Figure 2a,b show the starting material, intercalated graphite, before and during laser irradiation, respectively. Figure 2c–e is a closer demonstration of the expansion process. Figure 2c shows a single flake of intercalated graphite before laser irradiation, suspended in the air with the help of an acoustic levitation device, with a full image in Figure S3 (Camera setting and setup mentioned in the Measurements section). Figure 2d shows the flake while laser irradiates, while Fig. 2e shows the expanded flake after laser irradiation. Figure 2f presents the laser power threshold required for the expansion of intercalated graphite. “No expansion” refers to the condition where laser irradiation does not lead to any noticeable material expansion to the naked eye. “Slow expansion” (Supplementary video 2) indicates a gradual expansion of the graphite particles relative to a high-power output, whereas “Instant expansion” (Supplementary video 3) denotes the rapid evaporation of gas particles, causing the layers of graphite to expand quickly. It can be observed that approximately 2 watts of laser output is required to expand the intercalated graphite successfully. For a more detailed analysis, Fig. 2g focuses on the expansion in the current range of 0.4 to 0.6 amperes, confirming that the expansion of graphite particles can be accomplished with a laser output as low as 2 watts. To demonstrate the scalability of the present process, we utilized a commercial laser engraver, as depicted in Figure S4. As illustrated in Supplementary Video 1, the intercalated graphite undergoes expansion through the laser engraver, achieving laser-expanded graphite (LEG) with reduced time and minimal effort. Figure 3a shows that expansion volume and temperature rise as the laser output increases. Expansion remains minimal at low laser powers (0.2 W to 0.8 W). However, a sharp increase in expansion volume is observed between 1 and 2 W, suggesting that the laser has reached a localized temperature high enough to decompose perchloric acid particles. Beyond 2 W, expansion volume plateaus at around 800 mL g^−1^, indicating a saturation point where additional energy input does not significantly enhance expansion. The temperature, however, continues increasing linearly with power, exceeding 700 °C at the highest tested power (Figure S6). This suggests that while thermal effects continue, the expansion reaches a limit. Figure 3b shows Thermogravimetric analysis (TGA) revealed that LEG remains stable up to nearly 900 °C, meaning that the temperature increase in the experiment does not induce full decomposition or degradation.

The absorption of laser energy by graphite follows the photothermal effect ^84^, where incident photons interact with the electronic structure of the material, leading to rapid thermal energy conversion ^85^. When a laser beam strikes the graphite surface, its energy is absorbed by free electrons in the delocalized π-electron system of graphite, exciting them to higher energy states ^86–89^. These high-energy electrons then undergo electron–phonon coupling, transferring their excess energy to the atomic lattice as vibrational energy ^90–92^. This process leads to a localized and rapid temperature rise, which propagates through the material due to graphite’s high thermal conductivity ^90^. Upon laser irradiation, the intercalated perchloric acid undergoes thermal decomposition, releasing gases that expand the graphite structure ^91^:

$$4{\text{HClO}}_{4} \to 2{\text{Cl}}_{2} {\text{O}}_{7} + 2{\text{H}}_{2} {\text{O}}$$

$${\text{Cl}}_{2} {\text{O}}_{7} \to {\text{Cl}}_{2} + 3.5{\text{O}}_{2}$$

The rapid formation of O_2_, Cl_2_, and H_2_O vapor creates a high internal pressure between graphene layers, forcing expansion.

The continuous-wave laser irradiates the intercalated graphite from the top surface, and while a thermal gradient does form, the high thermal conductivity of graphite allows rapid heat dissipation. Additionally, the intercalation lowers the expansion temperature, enabling relatively uniform expansion through the thickness.

**Fig. 4:**
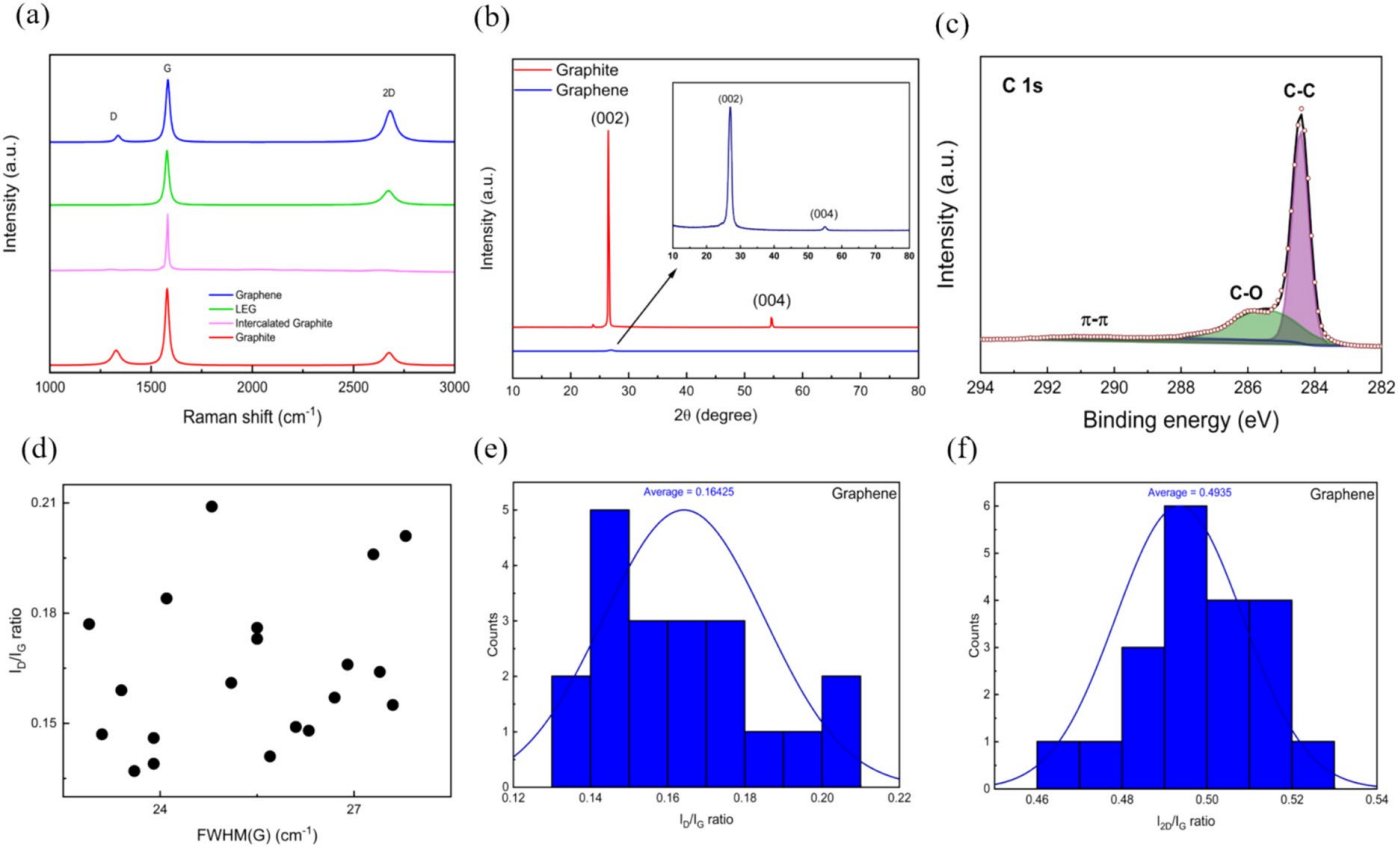
(**a**) Raman spectra of graphite, intercalated graphite, LEG, and graphene. (**b**) XRD patterns of graphite and graphene. (**c**) Convoluted fitting of XPS spectrum of C 1 s of graphene. (**d**) I_D_/I_G_ ratio as a function of FWHM(G) in graphene. (**e**) Statistical Histogram of I_D_/I_G_ ratio of graphene. (**f**) Statistical Histogram of I_2D_/I_G_ ratio of graphene. All are mapped across a 20 µm × 20 µm region.

X-ray diffraction (XRD) analysis was carried out on both the graphene and graphite to assess the crystallization degree of the synthesized graphene. In Fig. 4b, two characteristic XRD peaks at 2θ values of approximately 26.99° and 55.13° can be ascribed to the (002) and (004) crystallographic planes of graphite, respectively ^92^. The graphene XRD peaks are broader and exhibit reduced intensity compared to graphite ones. According to the calculations from Table S1, the Crystallite size (Dp) of graphite is 36.48 nm, whereas it is 7.53 for graphene. This reduction in crystallite size suggests a transformation in the material’s crystalline domains from larger graphite to smaller graphene sheets ^93^. XPS analysis was performed to evaluate the composition of graphene further. Figure S2b shows the survey spectra of graphene, in which only C and O can be observed. Figure 4c shows the C 1s spectra of graphene. Oxygen in graphene is evidenced by the O 1s peak observed in the XPS survey spectra. The intensity of this peak, relative to the C 1s peak, indicates the oxygen concentration on the graphene surface. The higher the O 1s peak intensity, the greater the oxygen content. The sharp and large carbon 1s (C1s) peak of graphene represented the graphitic carbon sp^2^ (C–C) and consisted of peaks corresponding to carbon bound (C–C) at ~ 284.4 eV and (C-O) at around 286 eV, respectively ^94^. However, for graphite, the carbon 1s (C1s) peak also prominently represents graphitic carbon sp^2^ (C–C) bonding but typically shows a slightly broader and less symmetric peak centered at approximately 284.5 eV, reflecting the multi-layered structure and varying electronic environments ^95^. Additionally, oxygen-containing functional groups are less pronounced, with any observable (C-O) bonding peaks around 286 eV; however, if they appear, they have generally lower intensity than graphene due to graphite’s bulk nature ^96^. Raman spectroscopy was done to identify distinctive peaks in graphite, intercalated graphite, expanded graphite, and graphene. The D Peak centered on ~ 1350 cm-1 manifests the structural defects in sheets arising due to disruption in regular aromatic sp2 networks that are so-called non-sp^2^ or sp^3^ defects ^97^. The G peak, located around 1580 cm⁻^1^, corresponds to the E_2g_ phonon at the Brillouin zone center of graphene ^98^. The 2D peak, observed at approximately 2700 cm⁻^1^, results from second-order Raman scattering by in-plane transverse optical phonons near the boundary of the Brillouin zone of graphene ^99^. Figure 4a shows the Raman spectra of graphite, intercalated graphite, LEG, and graphene. The Raman spectra of LEG flakes show peaks at 1348, 1586, and 2689 cm-1, corresponding to the D, G, and 2D bands, respectively. These bands are the most characteristic bands of a graphitic material ^100^. The effect of laser treatment appears in the significant decrease of the D peak, with an intensity ratio of the D and G bands (I_D_/I_G_) of NG 0.27, compared with LEG 0.11, the laser treatment is performed on intercalated graphite, leading to rapid thermal expansion and delamination rather than direct laser ablation of graphite basal planes. This process avoids excessive bond breaking and defect formation, preserving the sp^2^ carbon network. As a result, the D peak is negligible. Figure S2a illustrates the Raman spectrum of the intercalated graphite. It exhibits a peak at 1627 cm⁻^1^ (G peak), which, according to Liu, Daozhi, et al. ^101^, indicates that the intercalation stage is between stages 1 and 2. This peak serves as an indication of the successful intercalation process. As shown in Fig. 4a, the decreased intensity of the D band in graphene and the increased intensity of the 2D band compared to graphite indicates that it has few layer characteristics and fewer defects ^99^. The I_2D_/I_G_ ratio depends on the number of graphene layers ^102^. Typically, the I_2D_/I_G_ ratio is ~ 2–3 for monolayer graphene, 2 > I_2D_/_IG_ > 1 for bilayer graphene, and I_2D_/I_G_ < 1 for multilayer graphene ^103^. An I_2D_/I_G_ of ~ 0.5 is reported to correspond to a few-layered graphene (2–8 layers) ^104^. As shown in Fig. 4a, the I_2D_/I_G_ for graphite is ~ 0.33, while in Fig. 4f, I2D/I_G_ of graphene ranges from ~ 0.46 to ~ 0.53, averaging ~ 0.49 which indicates the presence of few to few-layer graphene and the success of the exfoliation process ^105^. It is essential to assess the quality of the obtained exfoliated graphene samples. As per the findings by AC Ferrari et al., ^106^ defect can be monitored by estimating the Full Width at Half Maximum (FWHM) of the G band, indicating that a broader G band is associated with a higher level of disorder. Additionally, the defect ratio, defined as the ratio of the intensity of the D band to the G band, is a crucial quantitative indicator for assessing the degree of disorder in graphene ^107^. To gain a deeper insight, it is essential to investigate the nature of defects, whether basal plane or edge defects. However, edge defects are inevitable since the ultrasonication process reduces flake sizes by causing fragmentation from kink bands ^106^. As shown in Fig. 4e, the statistical histograms for graphene samples’ I_D_/I_G_ ratios vary between approximately 0.13 and 0.21, with an average value of around 0.16. For further clarity, the correlation between the defect ratio (I_D_/I_G_) and FWHM of the G band has been plotted in Fig. 4d. The graph showed that values of FWHM of the G band are predominantly clustered in the range of 23–28 cm^-1^ for graphene. In other words, the range of defect ratios and the narrowed broadening of the G band in the graphene samples strongly suggest that the defects present are mainly due to edge defects, as opposed to defects in the basal plane.

**Fig. 5:**
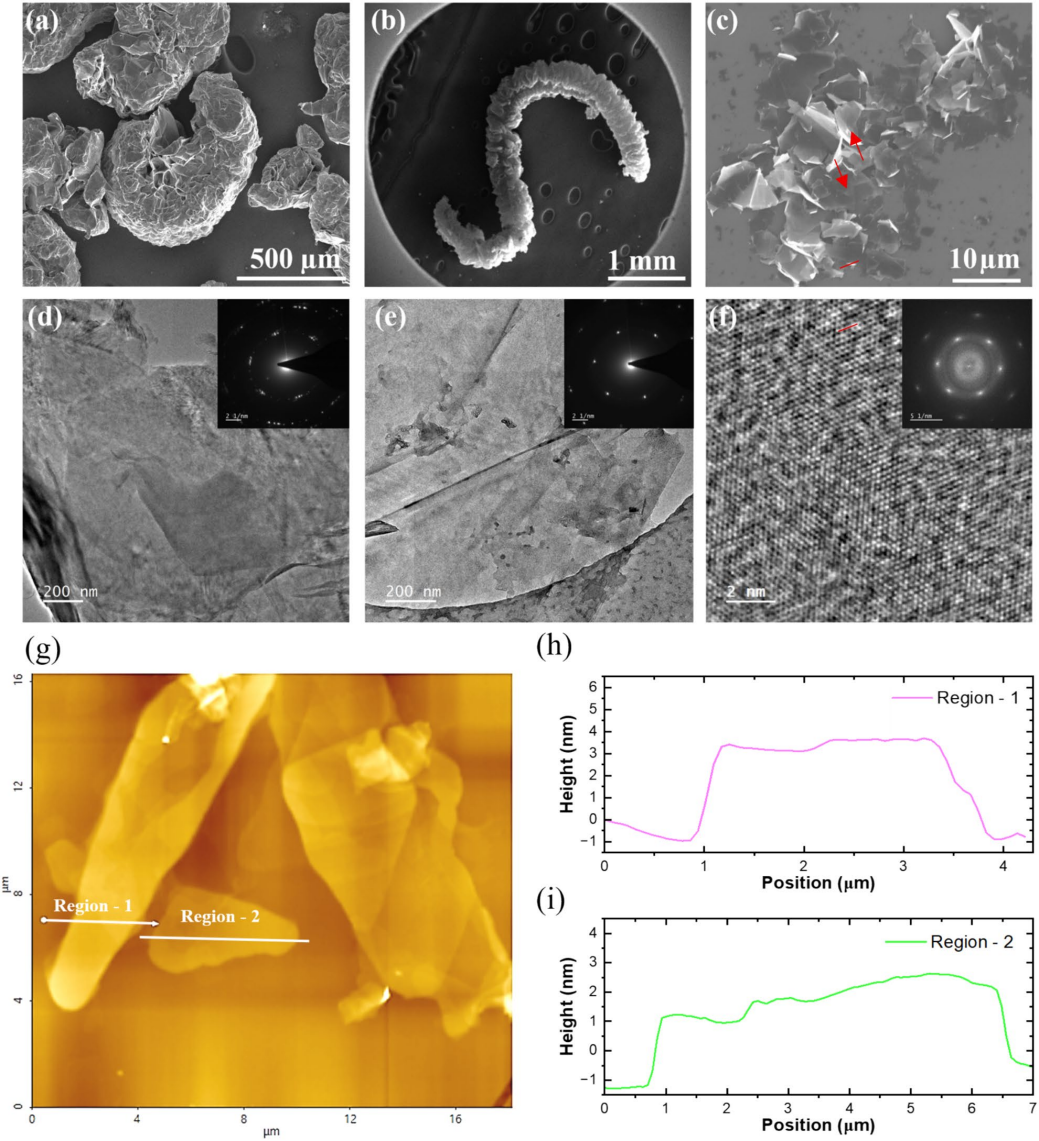
SEM at different magnifications of intercalated graphite (**a**), expanded graphite (**b**), and graphene (**c**). TEM of laser expanded graphite along with its SAED, LEG (**d**) Graphene (**e**) HRTEM of graphene (**f**) along with FFT. (**g**) AFM image of the graphene flakes. (**h**,**i**) Analysis of the thickness of the flakes in two different regions.

The scanning electron microscope (SEM) examined natural graphite (NG), GIC, LEG, and graphene morphological features. Figure S7a-c showed that graphite possessed a smooth surface, displaying no apparent porous characteristics. After undergoing intercalation treatment with perchloric acid, the layer spacing of GIC increased noticeably, resulting in an accordion-like structure with more expansive spaces (Fig. 5a). Furthermore, when GICs were subjected to laser irradiation, the intercalating species between the layers evaporated quickly, critical to the effective expansion of the graphite layers. Consequently, this process yields a worm-like structure, as shown in Fig. 5b. Figure 5c shows a highly dispersed array of graphene flakes with various shapes and sizes, suggesting a successful exfoliation process. EDX was used to analyze the elemental composition of the samples (Figure S1e-h). As shown in Figure S1f., the chlorine content increased significantly compared to graphite after the intercalation process, confirming the successful intercalation of the graphite sample. Figure S1g confirms the successful evaporation of the intercalation species, as the laser-expanded graphite contains no chlorine content and consists only of carbon. Figure S1h illustrates the known elemental composition of graphene, which consists of solely carbon atoms. Figure S1a-d shows the cross-sectional SEM of the free-standing graphene films. The cross-sectional SEM image displays the free-standing graphene films. As the added graphene volume to vacuum filtration increases, the thickness of the films also increases, ranging from approximately 11 to 69 µm. TEM and high-resolution TEM (HRTEM) analyses were conducted further to investigate the morphology and orientation of graphene samples. Figure 5d shows TEM images of LEG, showing multiple flakes stacked atop one another, consistent with the selected area electron diffraction (SAED) patterns that show relatively higher crystalline than graphene samples. Figure 5e shows a representative low-magnification TEM image of the graphene flakes. The lateral sizes of the flakes are relatively large, mainly in a few micrometers. Folded and wrinkled regions are observed in most of the flakes, and the SAED patterns of the sample consist of diffraction spots attributable to graphene ^108^. Figure 5f shows an HRTEM image, revealing a highly crystalline structure of the graphene with some defects due to exfoliation by ultrasonication. The Fast Fourier Transform (FFT) of the graphene shows a set of hexagonal patterns in the diffraction spots. Furthermore, Figure S2c shows the Electron Energy Loss Spectroscopy (EELS) for graphene. The C K-edge spectra of the graphene sample show an energy loss of around 284 eV. A clear feature of the peak at 284 eV is attributed to the C = C π* resonance, and the peak at 292 eV is attributed to the carbon sp^2^ bond (C–C σ* resonance) ^109^, which is consistent with the XPS spectrum in Fig. 4c. Figure 5g–i presents an analysis of graphene flakes using a high-resolution image with atomic force microscopy (AFM). Figure 5g shows a topographic image of graphene flakes on a silicon substrate, with two regions of interest, Region 1 and Region 2, marked for detailed analysis. Figure 5h,i illustrate the height profiles for the marked regions and provide insights into the layer thickness of graphene flakes. Graphene flake thickness ranges from 2 to 4 nm, indicating the presence of a few-layer graphene consistent with the Raman analysis.

**Fig. 6:**
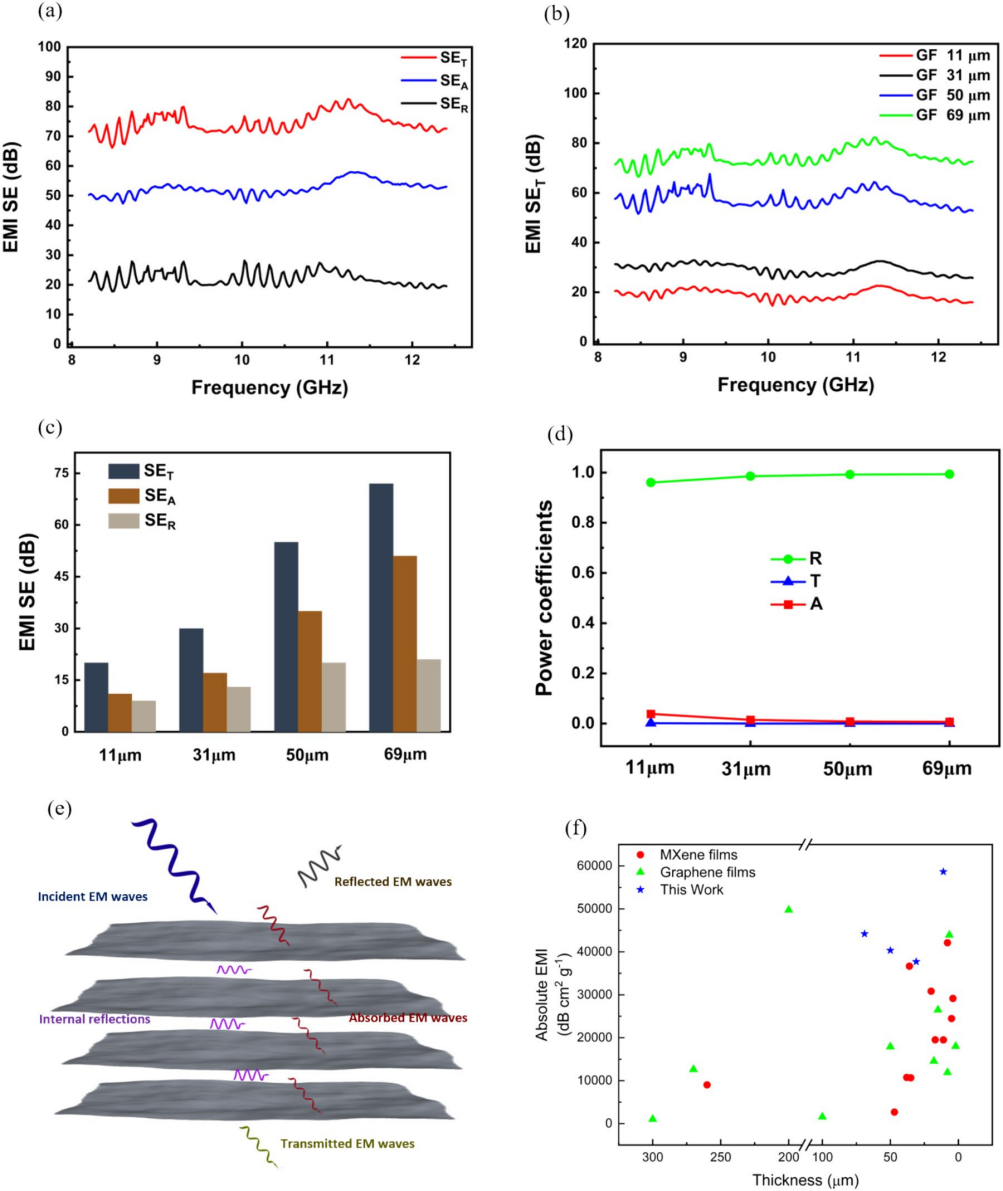
(**a**) SE_T_, SE_R_, and SE_A_ for GF-69 μm Films. (**b**) EMI SE is used for graphene films with various thicknesses. (**c**) SE_T_, SE_R_, and SE_A_ for graphene films with various thicknesses. (**d**) Power coefficients of reflection (R), transmission (T), and absorption (A) of graphene films as a function of thickness. (**e**) The possible EMI shielding mechanism of graphene films. (**f**) SSE/t of graphene films with different thicknesses compared with Graphene and MXene films from literature.

Electro Magnetic Interference shielding performance of graphene films was investigated in the X-band frequency range (8.2–12.4 GHz). Electromagnetic waves (EMWs) can be shielded by reflection, absorption, and multiple internal reflections ^110^. It is worth noting that the reflection of EMWs depends on the electrical conductivity of the shielding materials. At the same time, the absorption of EMWs is based on the electrical and magnetic dipoles of materials. In addition, multiple internal reflections are related to the abundant scattering centers and multiple interfaces in the structure of shielding materials ^111^. The graphene film with a thickness of 11 μm demonstrates sheet resistance of 1.9473 Ω/square, resistivity of 5.8418 µΩ m, and an excellent electrical conductivity of 1706.5 S cm^−1^. The thickness, density, and electrical conductivity of the films are mentioned in Table S2. Considering their optimal electrical properties, EMI shielding was measured in the X-band range for the graphene films. The total EMI (SE_T_), absorption (SE_A_), and reflection (SE_R_) of the Graphene film with a thickness of 69 μm are shown in Fig. 6a, delivering shielding efficiency values of approximately 72 dB, 51 dB, and 21 dB corresponding to SE_T_, SE_A_, and SE_R_ respectively. Notably, SE_R_ of the graphene film-69 μm presents a high value of nearly 21 dB, implying that the graphene film reflects more than 99% of EMWs. In this case, EMW reflection is dominant in the EMI shielding of graphene films. The remaining EMWs are further absorbed inside the film, contributing greatly to a super high value of SE_T_. Film thickness is believed to be crucial in EMI shielding performance ^112^. Therefore, graphene films with different thicknesses are successfully fabricated by changing the mass of the graphene. The EMI SE_T_ significantly increases with an increase in the thickness of the graphene films (Fig. 6b). The highest EMI SE_T_ is recorded at 72 dB for graphene film-69 μm. The thicker films provide a lengthened path for EMWs to travel, leading to plenty of interfaces and internal absorption and reflection, thus achieving more EMW consumption. Figure 5g,h are cross-sectional SEM images of the films with the lowest and highest thickness. Furthermore, the shielding behaviors of SEA and SER for graphene films with different thicknesses are plotted in Fig. 6c, revealing that SEA plays an important role in the shielding effectiveness for all graphene films. To further elucidate the EMI shielding mechanism, the power coefficients of reflection(R), absorption (A), and transmission (T) are calculated and summarized in Fig. 6d. Noticeably, the values of R (> 0.94) are much higher than those of A (< 0.06) for all graphene films, demonstrating that most incident EMWs are reflected at the interfaces of graphene films due to impedance mismatch and the reflection of EMWs dominates the shielding mechanism. A small quantity of remaining EMWs can enter the graphene film to be consumed by electromagnetic energy conversion. To sum up, the shielding process of the EMWs in graphene films is subject to a reflection-dominant shielding mechanism. The outstanding EMI shielding performance of the graphene films is derived from their high electrical conductivity and stacked porous architecture. The proposed mechanism, leading to the high EMI SE of graphene films, is shown in Fig. 6e. Specifically, most of the incident EM waves (> 90%) can be reflected when initially encountering the graphene films. This is owing to the interface impedance mismatch caused by the abundant free electrons on the surface of the conductive graphene films ^112^. After that, the remaining EMWs penetrate graphene films and interact with the high-density electron carriers (electrons and holes) in conduction paths for further dissipation ^113^. Meanwhile, the penetrated EMWs suffer from multiple internal reflections in the pores and the cavities of the graphene films, leading to further attenuation of EMWs. After the effective reflection and absorption, only a tiny amount of EMWs can pass through graphene films. High electrical conductivity and porous structure synergistically endow the graphene films with high-performance EMI SE. Specific shielding effectiveness and absolute effectiveness are other criteria used to estimate the potential application of EMI shielding materials, which can be calculated using Eqs. (7,8). All the graphene films (11 µm to 69 µm) present low densities of 0.310, 0.257, 0.273, and 0.236 g cm^−3^, respectively. Benefiting from the high electrical conductivity and low density, Graphene film -11 μm delivers the highest SSE/t of 58,666.7 dB cm^2^ g^−1^. With the increase of the thickness of the graphene film, the SSE/t diminishes to a lower value, which is probably owing to the enhanced EMI SE stemming from the longer EMW traveling path cannot compromise the increased thickness and weight of thicker graphene films, leading to lower SSE/t performance. The values of SSE/t for all the graphene films, along with Graphene and MXene films from recent literature, are calculated and presented in Fig. 6f and Table S3. MXene has emerged as a material of significant interest due to its potential for EMI shielding applications ^102,103^. While MXenes exhibit higher electrical conductivity, which is a favorable attribute for EMI shielding, they tend to have a lower absolute EMI shielding efficiency than the present work. This lower efficiency can be attributed to the higher weight of MXene composites compared to graphene films ^114,115^.

## Conclusion

In this work, an innovative approach was developed to synthesize graphene utilizing a laser through the expansion of graphite with a simple preparation process for effective EMI shielding efficiency. Under optimally tuned laser conditions, LEG is efficiently produced using a mere two Watts of output power, completing expansion up to 800 mL g^−1^ in seconds. The present findings reveal that free-standing graphene produced from LEG exhibits exceptional electrical conductivity of 1706 S cm^−1^, EMI shielding efficiency of 72 dB for 69 µm thick free standing graphene film, and a remarkable absolute shielding effectiveness of 58,666 dB cm^2^ g^−2^ for 11 µm thickness film. A comprehensive suite of characterization techniques was employed to investigate the morphology and structure of the samples systematically. Raman spectroscopy analysis confirmed the high quality of the graphene produced, as evidenced by an I_D_/I_G_ ratio of approximately 0.13 and an I_2D_/I_G_ ratio of around 0.52. Further analyses of AFM and HRTEM revealed that the graphene sheets predominantly exhibit a folded morphology, with few-layer graphene sheets entangling each other, consistent with the Raman data.

## Experimental section

### Chemicals and reagents

Graphite flakes (average size of 100 μm), perchloric acid (HClO_4_, 70 wt.%), deionized water, and isopropyl alcohol (C_3_H_8_O, 99%) were purchased from Sigma-Aldrich. All the chemicals used in the experiments were used without further purification.

### Synthesis of expanded graphite

Typically, 1 g of graphite flakes and 2.5 mL of perchloric acid were mixed in a weight ratio 1:4 for 30 min at 150 °C to form a graphite intercalated compound. GIC was taken into a porcelain dish and placed in the laser setup mentioned in Fig. 1a, with a 2 cm distance from the CW-blue laser source. After being irradiated under the laser for 30 s, LEG was collected.

### Synthesis of graphene and graphene films

LEG was then exfoliated in isopropyl alcohol by sonication using a probe sonicator for 1 h. The supernatant with exfoliated graphene was collected after centrifuging at 900 RPM for 45 min.

Desired amounts of the exfoliated graphene suspension were filtrated under a vacuum to form free-standing graphene films with different thicknesses.

### Characterization and measurements

The crystallinity of the samples was measured using X-ray diffraction (XRD Bruker D2 phaser). Raman analysis of the starting materials and the exfoliated samples were performed by Renishaw spectrometer using 532 nm laser (2.33 eV) excitation and 50 × objective lens. The laser power was kept below 1 mW to prevent sample damage. 20 spectra were recorded (each one at a different location) for each sample to create statistical data for the samples. Morphology of the samples was done by scanning electron microscopy ((SEM) Nova NanoSEM 650, beam resolution 0.8 nm). AFM analysis was done using the Park system FX50 module. XPS spectra were recorded using a Thermo Scientific K-Alpha spectrometer. Intercalated graphite samples were irradiated using a CW-blue laser with a wavelength of 455 nm, focus length of 40 mm, spot size of 0.16–0.18 mm, and output optical power of 20 W. Digital images were taken using a Sony Alpha A6400 camera with an 18 Mm lens, 24.2 megapixels, and iso was adjusted to 100. Graphite samples were suspended in the air using a DC 12Volts ultrasonic levitation device. The graphite’s expansion volume (EV) was calculated by equation ^72^. EG was moved into a measuring cylinder for volume testing, and the average value was calculated after removing the maximum and minimum values, considering the accuracy of the testing process.

1 $${\text{EV}} = {\text{V}}/{\text{m}}$$

where EV is the graphite expansion volume, mL g^−1^; V is the volume after expansion, mL; m is the mass of the specimen, g.

EMI shielding performance of graphene films was measured using a 2-port network analyzer (ENA5071C, Agilent Technologies, USA) in the X-band frequency range (8.2–12.4 GHz). All the graphene film samples were cut into a rectangular shape, slightly larger than the sample holder, to avoid any leakage paths from the edges. Electromagnetic radiation includes reflection (R), absorption (A), and transmission (T) (A + R + T = 1). The total EMI shielding effectiveness (SE_T_), reflection effectiveness (SE_R_), and absorption effectiveness (SE_A_) were calculated following equations below:

2 $$R={|{S}_{11}|}^{2}={|{S}_{22}|}^{2}$$

3 $$T={|{S}_{12}|}^{2}={|{S}_{21}|}^{2}$$

4 $${SE}_{T}={SE}_{R}+{SE}_{A}$$

5 $${SE}_{R}=10\text{log}\left(\frac{1}{1-R}\right)=10\text{log}\left(\frac{1}{1-{|{S}_{11}|}^{2}}\right)$$

6 $${SE}_{A}=10\text{log}\left(\frac{1-R}{T}\right)=10\text{log}\left(\frac{1-{|{S}_{11}|}^{2}}{{|{S}_{21}|}^{2}}\right)$$

Taking the thickness and density of films into account, the Specific shielding effectiveness (SSE) and Absolute effectiveness (SSE/t) can be calculated using the following equation:

7 $$SSE=SE/density (dB {cm}^{3}{ g}^{-1})$$

8 $$SSE/t=SE/(density\times thickness)(dB {cm}^{2}{ g}^{-1})$$

Electrical characterization of the samples was performed on thin films prepared from the dispersions. Sheet resistance measurements were carried out by four-point probe technique using Ossila Four-Point Probe system. The conductivity values of the thin films were calculated from the measured sheet resistances using Eq. (9).

9 $$\sigma_{el} = \, Rs^{ - 1} \times \, t^{ - 1}$$

where σ_el_ is the electrical conductivity; Rs is the sheet resistance and t is the thickness of the film.

Raman fitting analysis was done using Lorentzian equation which is shown below.

10 $$y={y}_{o}+\frac{2A}{\pi } \frac{w}{4\left(x-{x}_{c}^{2}\right)+{w}^{2}}$$

## Supplementary Information

Supplementary Information 1.

Supplementary Video 1.

Supplementary Video 2.

Supplementary Video 3.

## Data Availability

Data is provided within the manuscript or supplementary information files.
