# Supplementary material for: Unprecedented expansion of graphite with low power laser for high-quality liquid phase exfoliated graphene
Source: Sci Rep. 2025 Sep 24;15:32733. doi: 10.1038/s41598-025-17947-6 (PMC12460847; doi:10.1038/s41598-025-17947-6)
Supplement: Supplementary file 1 — Supplementary Information 1. [file 41598_2025_17947_MOESM1_ESM.docx]

**Supplementary Information**

**Unprecedented Expansion of Graphite With Low Power Laser For High-Quality Liquid Phase Exfoliated Graphene**


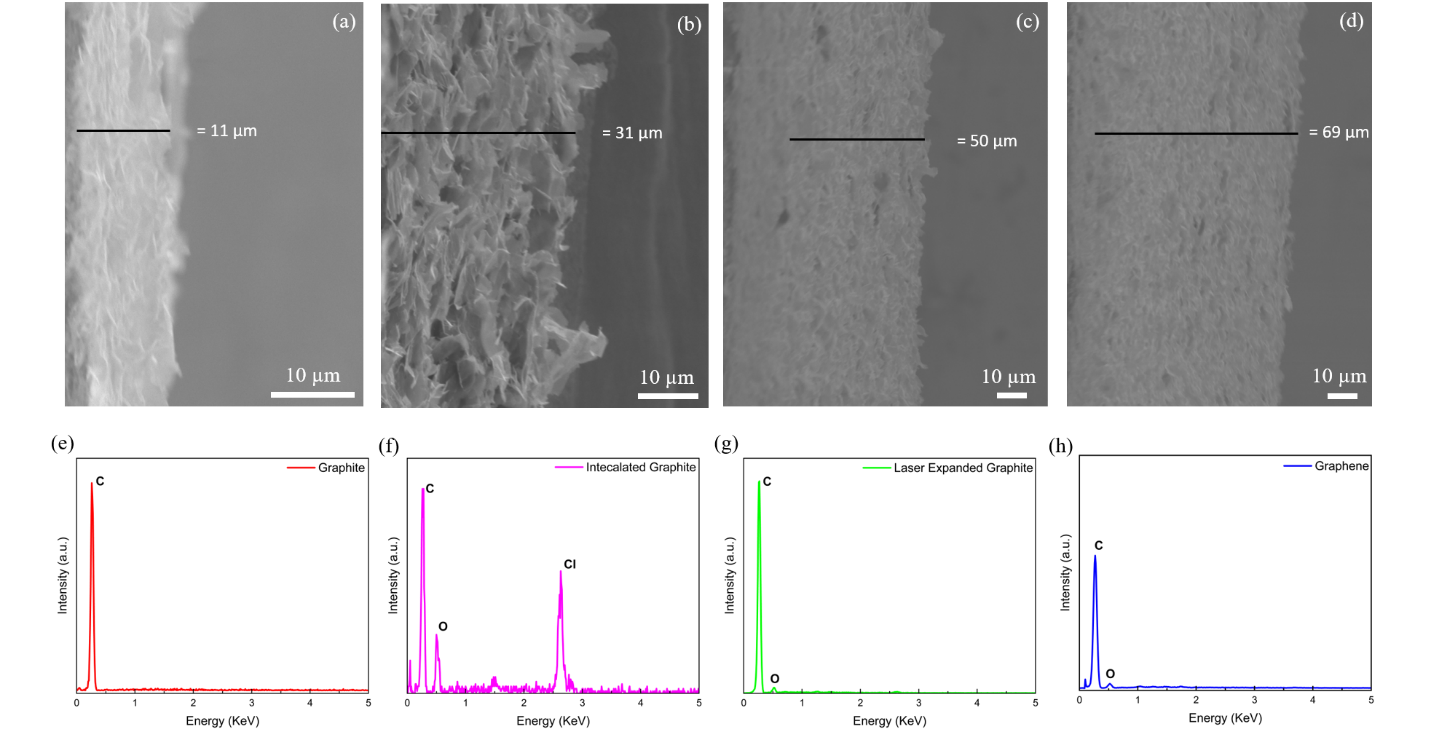


Figure S1. Cross sectional SEM of graphene films (a) 11 µm. (b) 31 µm. (c) 50 µm. (d) 69 µm. EDX of (e) Graphite. (f) Intercalated graphite. (g) Laser expanded graphite. (h) Graphene.


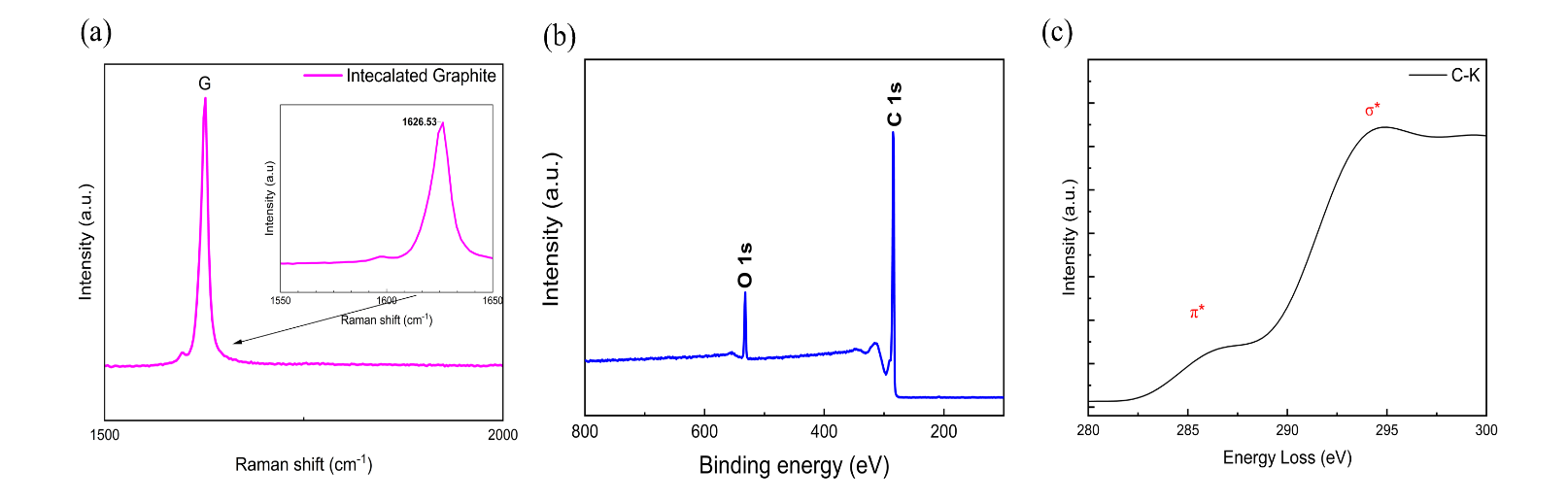


Figure S2. (a) Raman spectrum of intercalated graphite. (b) XPS survey spectrum of graphene. (c) EELS of graphene.


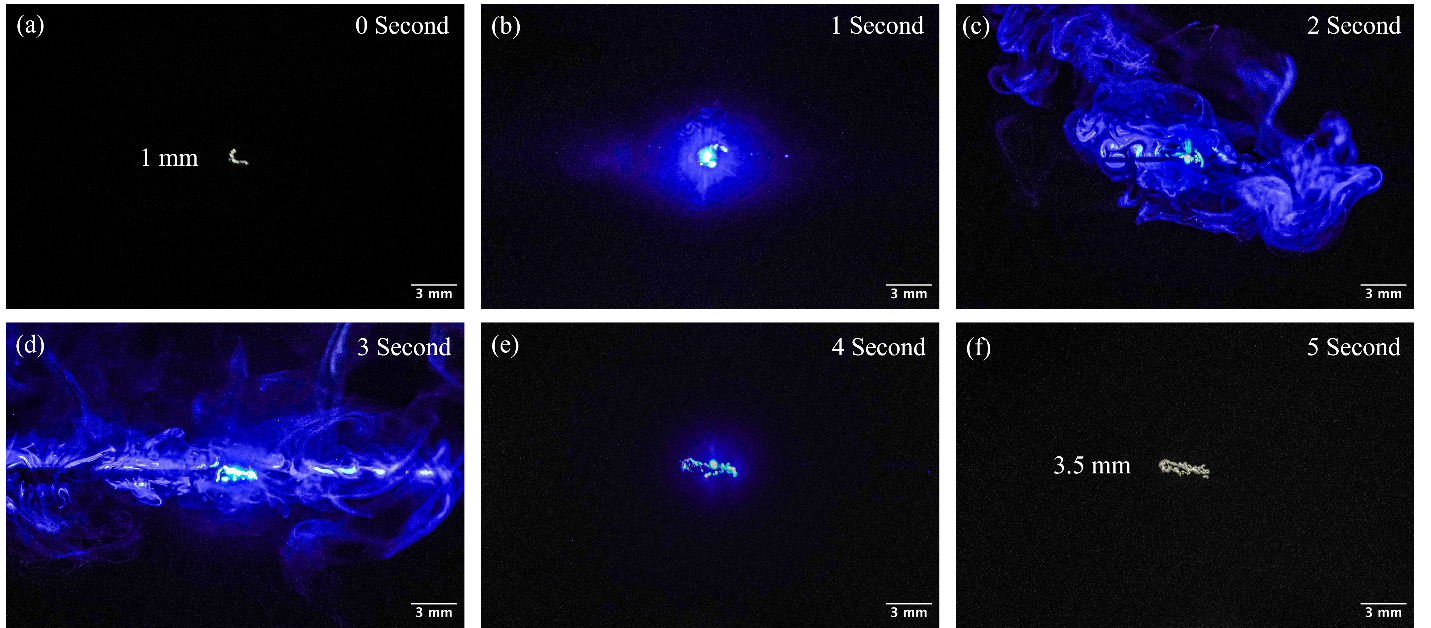


Figure S3. Laser expansion of single flake (a) before irradiation. (b-e) While irradiation. (f) After irradiation.


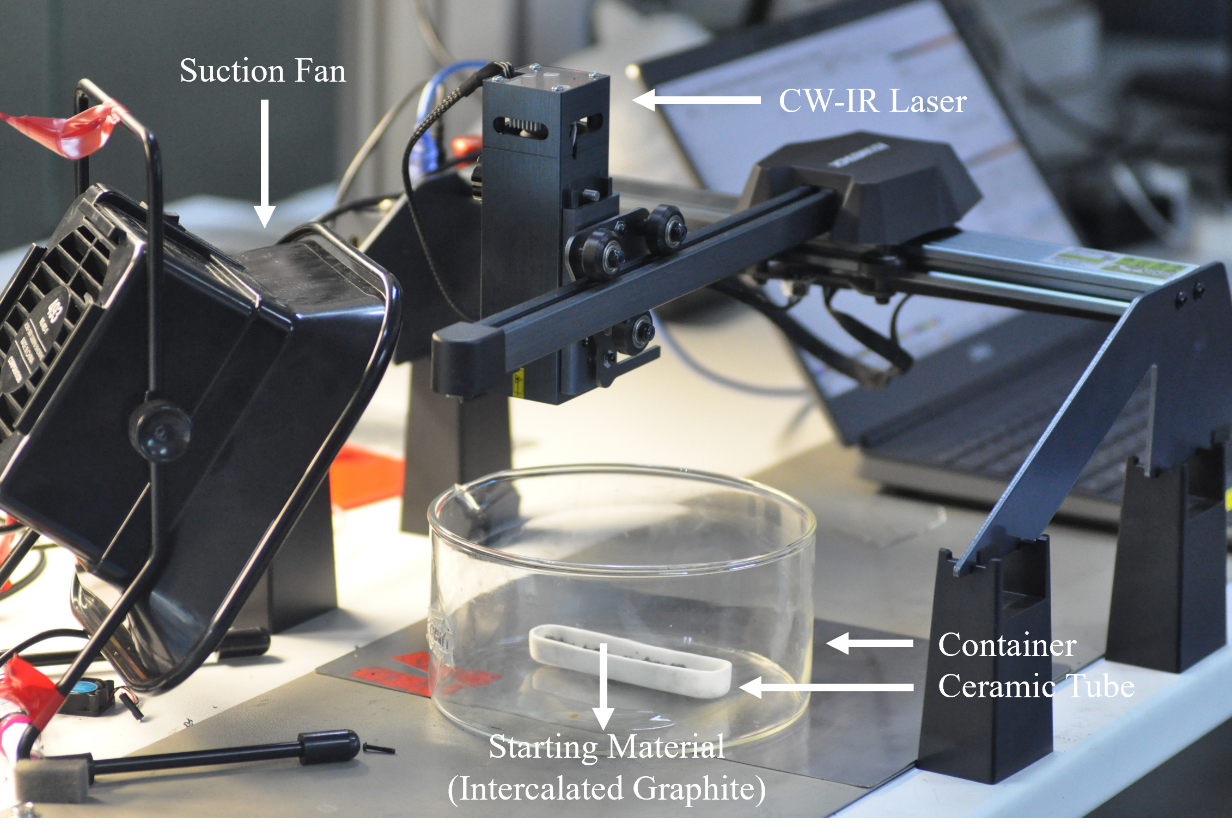


Figure S4. Laser setup for graphite expansion.


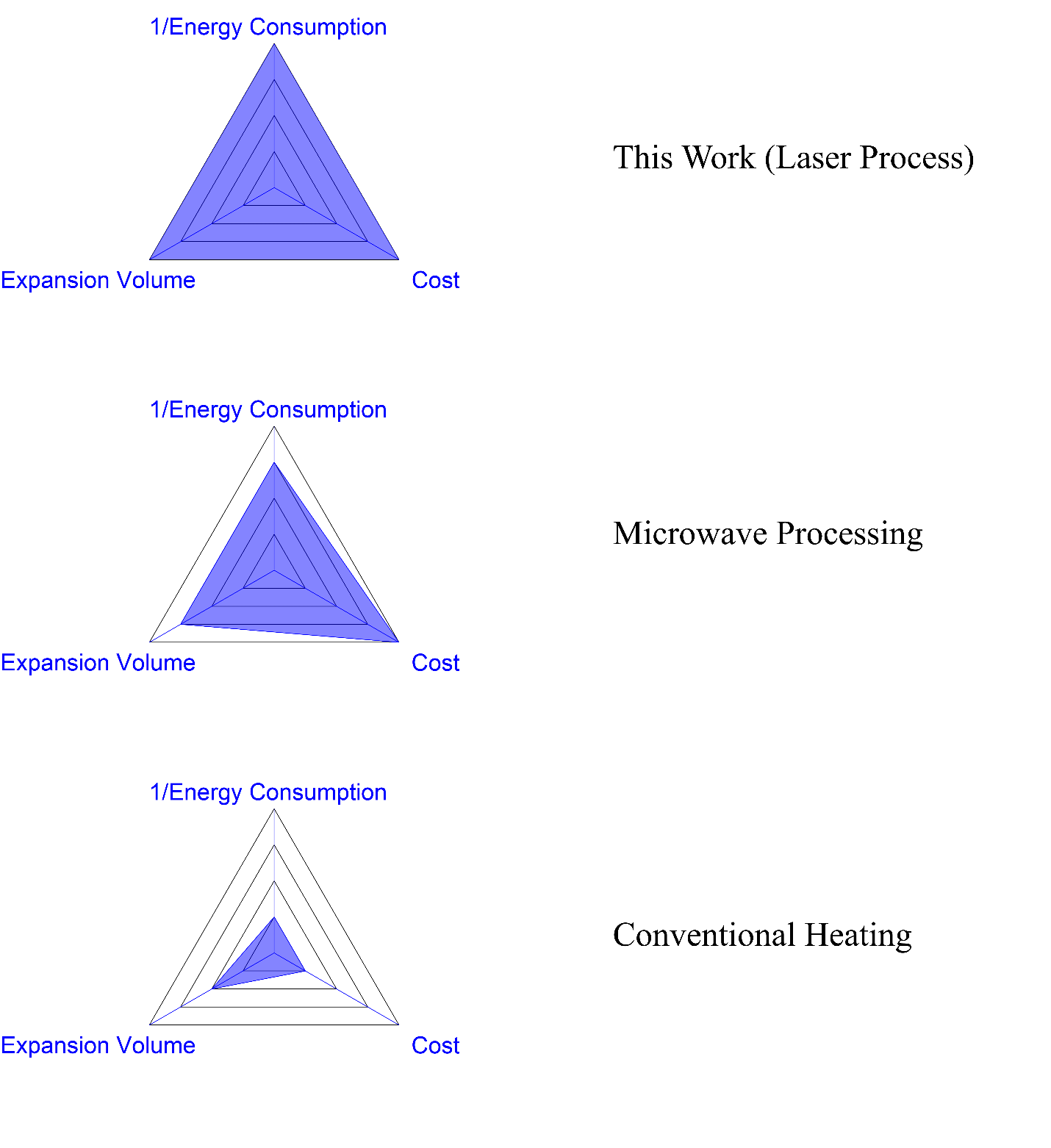


Figure S5. Comparison between laser, microwave, and conventional heating processes based on the data from Tables S4 and S5.

The three methods—laser, microwave, and conventional heating—exhibit significant differences in expansion volume, cost, and energy efficiency. To produce 1g of LEG, the laser method is the best performer, achieving the highest expansion volume while maintaining the lowest energy consumption at just 0.0005 kWh, making it 2,400 times more efficient than conventional heating and 23 times more efficient than microwave processing. Its cost is also relatively low, making it the most optimal choice for material expansion. The microwave method, while not as efficient as laser, still provides a decent expansion volume with moderate cost and significantly lower energy consumption (0.01167 kWh) compared to conventional heating. However, it is still less energy-efficient than laser processing. The conventional heating method, on the other hand, has the lowest expansion volume, highest cost, and extremely high energy consumption at 1.2 kWh, making it the least desirable method for material processing.

In contrast to the former conventional methods, the present laser expansion of graphite offers several key advantages. It is more energy-efficient than thermal or microwave expansion, requiring only a two-watt laser output. Çalın Ö et al. [1] demonstrated that slow heating during the thermal expansion process was not significant in expanding graphite layers because the process of graphite expansion was mainly dependent on the extreme forces created by a rapid gas explosion, which comes in favor of this present process as it relies on the instant and localized heat from the laser source. The laser heats specific areas of the graphite to high temperatures almost instantaneously, leading to rapid expansion.


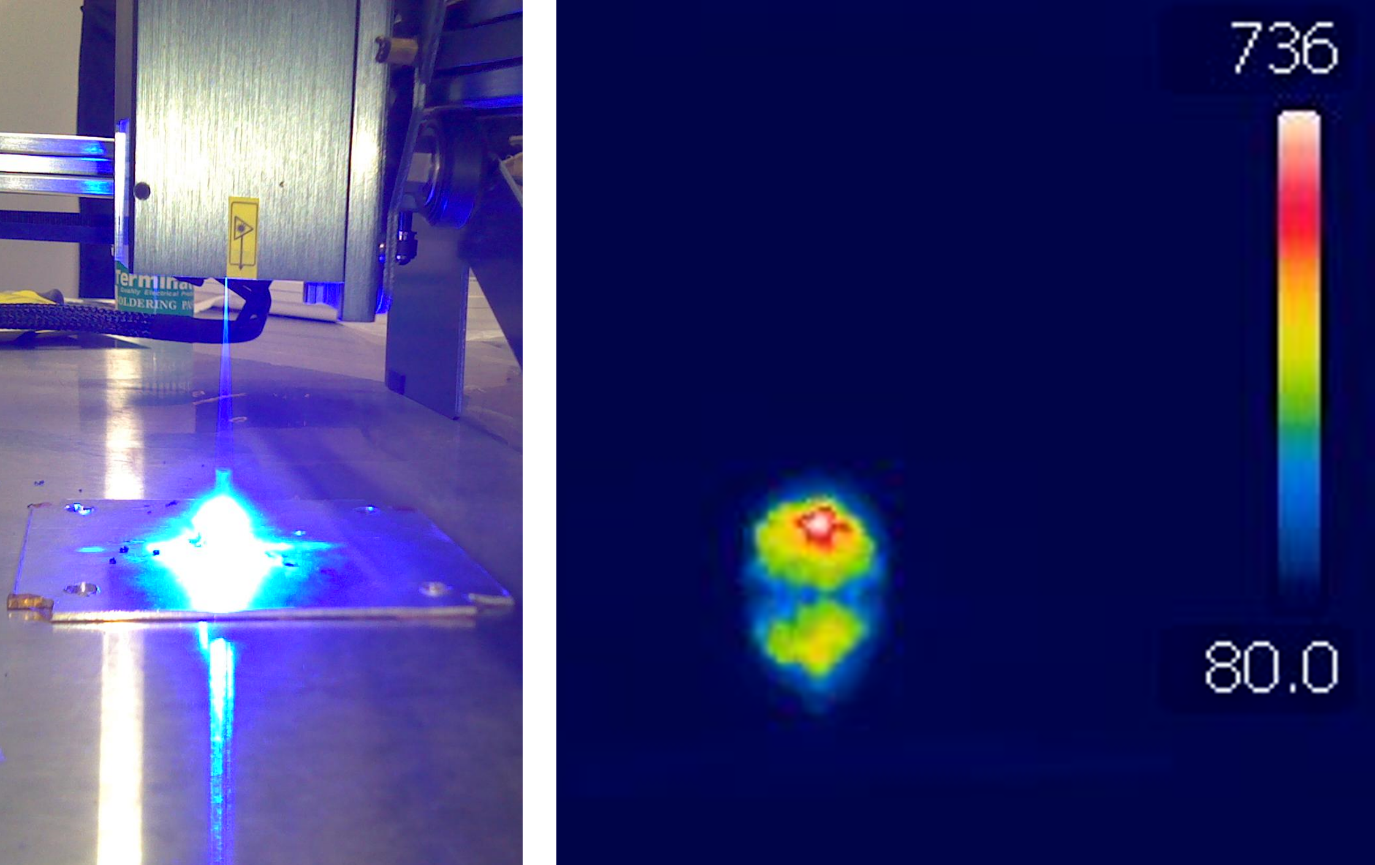


Figure S6. calculated the Localized temperature of the laser process using a thermal camera.


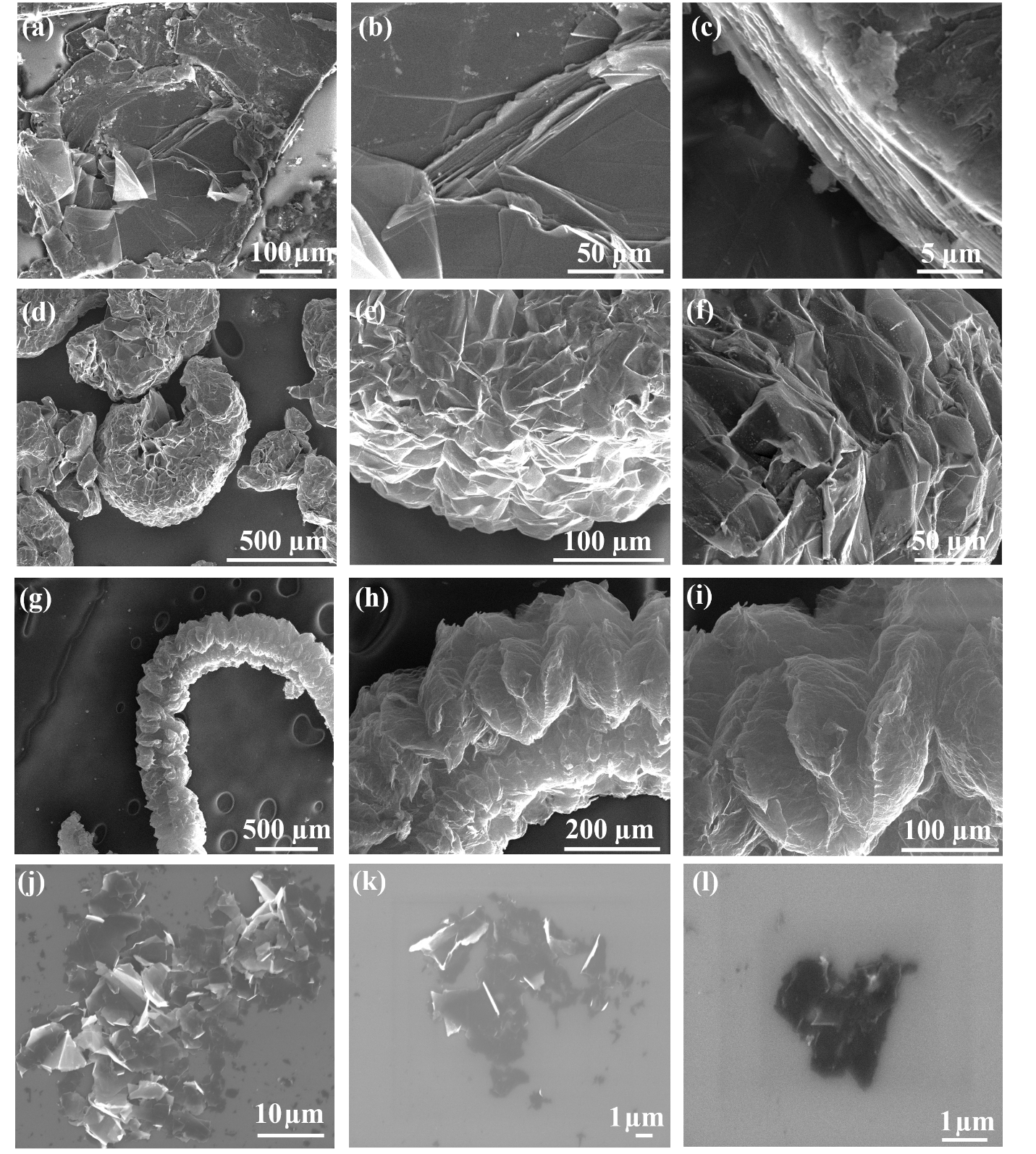


Figure S7. SEM at different magnification of graphite (a-c), Intercalated graphite (d-f), Expanded graphite (g-i), Graphene (j-l).

**XRD**

Scherrer's Formula [2]:

Crystallite size (Dp) = K λ / (β cos θ)

K - Scherrer constant. K varies from 0.68 to 2.08.

K = 0.94 for spherical crystallites with cubic symmetry.

λ - X-ray wavelength. For XRD, Cu Kα average = 1.54178 Å

|  | 2θ | FWHM | Dp (nm) | d-spacing |
| --- | --- | --- | --- | --- |
| Graphite | 26.99 | 0.23406 | 36.48 | 3.3 |
| Graphene | 26.55 | 1.13371 | 7.53 | 3.35 |

β - FWHM (Full Width at Half Maximum) of XRD peak.

**Supplementary Table 1. Date obtained about crystal structure of Graphite and graphene from XRD.**

**Supplementary Table 2. Parameters of graphene films.**

|  | Weight  (mg) | Thickness  (μm) | Density  (g/cm^3^) | Conductivity (S/cm) | EMI Shielding  (dB) | Specific EMI  (dB cm^3^ g^-1^) |
| --- | --- | --- | --- | --- | --- | --- |
| Graphene film- 11 µm | 0.9 | 11 | 0.310 | 1706.537 | 20 | 64.533 |
| Graphene film- 31 µm | 2.1 | 31 | 0.257 | 410.924 | 30 | 116.914 |
| Graphene film- 50 µm | 3.6 | 50 | 0.273 | 290.467 | 55 | 201.667 |
| Graphene film- 69 µm | 4.3 | 69 | 0.236 | 200.624 | 72 | 305.012 |

**Supplementary Table 3. Current work compared with data obtained from literature for MXene.**

| Filler/Matrix | Thickness  (μm) | Conductivity (S/cm) | EMI Shielding  (dB) | Absolute EMI  (dB cm^2^ g^-1^) | Refs |
| --- | --- | --- | --- | --- | --- |
| Ti_3_AlC_2_/CNF | 47 | 749.4 | 25.8 | 2674 | [3] |
| Ti_3_C_2_T_x_ /rGO SNF/PVA | 260 | 13.3 | 55.81 | 9000 | [4] |
| Ti_3_C_2_T_x_ /CS | 35 | 9.69 | 40.8 | 10650 | [5] |
| Ti_3_C_2_T_x_ /CNF | 38 | 466.85 | 50.6 | 10727.7 | [6] |
| Ti_3_AlC_2_/PEDOT: PSS | 11 | 340.5 | 42.1 | 19497 | [7] |
| Ti_3_C_2_T_x_ /PVDF | 17 | 214.6 | 42.9 | 19504.8 | [8] |
| Ti_3_C_2_T_x_ / Xanthan | 5 | 115.30 | 48 | 24464.8 | [9] |
| Ti_3_C_2_T_x_ /BC | 4 | 156.25 | 37.7 | 29141 | [10] |
| Ti_3_C_2_T_x_ /ANF | 20 | - | 57.19 | 36641.94 | [11] |
| CNT/Ti_3_C_2_T_x_ | 36 | 341.44 | 70 | 42109 | [12] |
| Ti_3_C_2_T_x_/SA | 8 | 3000 | 57 | 30830 | [13] |
| rGO | 2 | 100 | 33.50 | 18000 | [14] |
| rGO-EDA | 6.6 | 8796 | 58.5 | 43902 | [15] |
| GO | 200 | 25 | 63.0 | 49750 | [16] |
| GO | 8 | 1000 | 20 | 11904 | [17] |
| Graphene/Fe_3_O_4_ | 300 | - | 24 | 1033 | [18] |
| Graphene | 50 | 714 | 60 | 17910 | [19] |
| Graphene | 18 | - | 55 | 14550 | [20] |
| Graphene/CNT | 15 | 2740 | 57.6 | 26483 | [21] |
| Graphene/CNT/PVDF | 100 | 0.012 | 27.58 | 1557 | [22] |
| rGO/Fe_3_O_4_/SiO_2_ | 270 | 0.71 | 32 | 12608.4 | [23] |
| **Graphene film- 31 µm** | 31 | 410.924 | 30 | **37714.286** | **ThisWork** |
| **Graphene film- 50 µm** | 50 | 290.467 | 55 | **40333.333** |  |
| **Graphene film- 69 µm** | 69 | 200.624 | 72 | **44204.651** |  |
| **Graphene film- 11 µm** | 11 | 1706.537 | 20 | **58666.667** |  |

Abbreviations: CNF-cellulose nanofiber, ANF-aramid nanofiber, BC-bacterial Cellulose, rGO-reduced graphene oxide, PVDF- polyvinylidene fluoride, CNT-carbon nanotubes, CS-Chitosan, SNF- spunlace non-woben fabric, PVA-polyvinyl alcohol, SA- Sodium alginate. EDA- ethylenediamine

**Supplementary Table 4 Analysis of laser, microwave, and conventional heating processes.**

| Method | Equipment used | Power | Average Time | Total Electricity Consumed in (kWh) | Cost of the machinery |
| --- | --- | --- | --- | --- | --- |
| Laser process of 1g of EG | Laser Module | 2W | 1 minute | 0.0005 | Averaging 100 USD |
|  | Laser Power Supply | 5W |  |  |  |
|  | Control Board | 5W |  |  |  |
|  | Stepper Motor (X & Y axis) | 10W |  |  |  |
|  | Cooling System | 3W |  |  |  |
| Microwave process of 1g of EG | Domestic Microwave | 700W | 1 minute | 0.01167 | Averaging 100 USD |
| Conventional Heating process of 1g of EG | Oven | 1200W | 1 hour | 1.2 | Averaging 1000 USD |

Electricity consumed = Device power consumption (W) × time of use (h)

**Supplementary Table 5 Literature analysis of the laser, microwave, and conventional heating processes.**

| Method | Intercalation Conditions | | | Expansion Conditions | | Expansion Volume (mL/g) | Ref |
| --- | --- | --- | --- | --- | --- | --- | --- |
|  | Reagents | Temperature | Time | Heat/Watt | Time |  |  |
| Microwave | HNO_3_/KMnO_4_ | RT | NA | 700 W | 60s | 312 | [24] |
|  | KMnO_4_/HClO_4_ | RT | 10s | 600W | 50s | 560 | [25] |
|  | HClO_4_/NaNO_3_ | RT | 10s | 800W | 50s | 534 | [26] |
|  | K_2_S_2_O_8_ H_2_SO_4_ | 50 ˚C | 300s | 800W | 40s | 455 | [27] |
|  | HClO_4_ | RT | 10 s | 800 W | 50-60 s | 524 | [28] |
|  | KMnO_4_ H_2_SO_4_ | 75 ˚C | 30 min | 700 W | 10 s | 9.73 | [29] |
|  | HClO_4_ HNO_3_ H_3_PO_4_ KMnO_4_ CTAB KBr | NM | 4h | 800 W | 20s | 390 | [30] |
|  | KMnO_4_/HClO_4_ | 80 ˚C | 12h | 750W | 20s | 760 | [31] |
|  | HNO_3_/KMnO_4_ | RT | 3min | 700W | 60s | 317 | [32] |
|  | (NH_4_)_2_S_2_O_8_ H_2_SO_4_ | 60 ˚C | 6h | 800W | 120s | 267 | [33] |
|  | KMnO_4_/HClO_4_ | RT | 10s | 600W | 50s | 565 | [34] |
|  | NaClO_4_/C_2_H_2_O_4_ | RT | 60min | NA | NA | 290 | [35] |
| Conventional Heating | HNO_3_/HClO_4_ | NA | NA | 900˚C | 30s | 360 | [36] |
|  | (NH_4_)_2_S_2_O_8_ H_2_SO_4_ | RT | 12h | Room Temperature | 12h | 225 | [37] |
|  | NO_2_ (g) | 55 ˚C | 48h | 1000 ˚C |  | 240 | [38] |
|  | H_2_O_2_/HClO_4_/KMnO_4_/HAc | NA | 7h | 300 ˚C | 1h | 320 | [39] |
|  | KMnO_4_/NH_4_NO_3_/HCl | 30 ˚C | 10 min | 900 ˚C | 300 s | 480 | [40] |
|  | HNO_3_/C_3_H_6_O_2_/CH_3_COOH | RT | 20 min | 900 ˚C | 120 s | 378 | [41] |
|  | KMnO_4_ H_2_SO_4_ | RT | 15min | 950 ˚C | 8 s | 400 | [42] |
|  | H_2_SO_4_ HNO_3_ | NA | 1.5h | NM | 1h | 149 | [43] |
|  | KMnO_4_ H_2_SO_4_ H_2_O_2_ | RT | NA | RT | NA | 250 | [44] |
|  | HNO_3_/HClO_4_ | 35 ˚C | 50min | 800 ˚C | NA | 241 | [45] |
|  | H_2_SO_4_/Na_2_S_2_O_8_ | RT | 30min | NA | NA | 140 | [46] |
|  | K_2_Cr_2_O_7_/HClO_4_ | 45 ˚C | 1h | 200 ˚C | 2h | 350 | [47] |
|  | KMnO_4_/HClO_4_ | 40 ˚C | 1h | 950 ˚C | 15s | 420 | [48] |
|  | H_2_SO_4_/K_2_S_2_O_8_ | 80 ˚C | 300 s | NA | NA | 150 | [49] |
|  | NA | NA | NA | 300 ˚C | NA | 280 | [50] |
|  | HClO_4_ | NA | NA | 900 ˚C | 30s | 360 | [51] |
| Laser Process | H_2_SO_4_ | NA | NA | NA | NA | NA | [52] |
|  | NA | NA | NA | NA | NA | NA | [53] |
|  | NA | NA | NA | NA | NA | NA | [54] |
|  | **HClO_4_** | **150 ˚C** | **30min** | **2 Watt-Laser** | **60s** | **800** | **This Work** |

**References**

[1] Ö. Çalın, A. Kurt, and Y. Çelik, “Influence of expansion conditions and precursor flake size on porous structure of expanded graphite,” *Fuller. Nanotub. Carbon Nanostructures*, vol. 28, no. 8, pp. 611–620, Aug. 2020, doi: 10.1080/1536383X.2020.1726894.

[2] A. Monshi, M. R. Foroughi, and M. R. Monshi, “Modified Scherrer Equation to Estimate More Accurately Nano-Crystallite Size Using XRD,” *World J. Nano Sci. Eng.*, vol. 02, no. 03, pp. 154–160, 2012, doi: 10.4236/wjnse.2012.23020.

[3] W.-T. Cao *et al.*, “Binary Strengthening and Toughening of MXene/Cellulose Nanofiber Composite Paper with Nacre-Inspired Structure and Superior Electromagnetic Interference Shielding Properties,” *ACS Nano*, vol. 12, no. 5, pp. 4583–4593, May 2018, doi: 10.1021/acsnano.8b00997.

[4] Y. Zhang *et al.*, “rGO/MXene sandwich-structured film at spunlace non-woven fabric substrate: Application to EMI shielding and electrical heating,” *J. Colloid Interface Sci.*, vol. 614, pp. 194–204, May 2022, doi: 10.1016/j.jcis.2022.01.030.

[5] Z. Tan *et al.*, “Fabrication of Chitosan/MXene multilayered film based on layer-by-layer assembly: Toward enhanced electromagnetic interference shielding and thermal management capacity,” *Compos. Part Appl. Sci. Manuf.*, vol. 155, p. 106809, Apr. 2022, doi: 10.1016/j.compositesa.2022.106809.

[6] S. Feng, Y. Yi, B. Chen, P. Deng, Z. Zhou, and C. Lu, “Rheology-Guided Assembly of a Highly Aligned MXene/Cellulose Nanofiber Composite Film for High-Performance Electromagnetic Interference Shielding and Infrared Stealth,” *ACS Appl. Mater. Interfaces*, vol. 14, no. 31, pp. 36060–36070, Aug. 2022, doi: 10.1021/acsami.2c11292.

[7] R. Liu, M. Miao, Y. Li, J. Zhang, S. Cao, and X. Feng, “Ultrathin Biomimetic Polymeric Ti _3_ C _2_ T *_x_* MXene Composite Films for Electromagnetic Interference Shielding,” *ACS Appl. Mater. Interfaces*, vol. 10, no. 51, pp. 44787–44795, Dec. 2018, doi: 10.1021/acsami.8b18347.

[8] Y. Li *et al.*, “Scalable manufacturing of flexible, durable Ti3C2Tx MXene/Polyvinylidene fluoride film for multifunctional electromagnetic interference shielding and electro/photo-thermal conversion applications,” *Compos. Part B Eng.*, vol. 217, p. 108902, Jul. 2021, doi: 10.1016/j.compositesb.2021.108902.

[9] Y. Sun *et al.*, “MXene-xanthan nanocomposite films with layered microstructure for electromagnetic interference shielding and Joule heating,” *Chem. Eng. J.*, vol. 410, p. 128348, Apr. 2021, doi: 10.1016/j.cej.2020.128348.

[10] Y. Wan *et al.*, “Ultrathin, Strong, and Highly Flexible Ti _3_ C _2_ T *_x_* MXene/Bacterial Cellulose Composite Films for High-Performance Electromagnetic Interference Shielding,” *ACS Nano*, vol. 15, no. 5, pp. 8439–8449, May 2021, doi: 10.1021/acsnano.0c10666.

[11] J. Lu *et al.*, “Ultrathin and Mechanically Robust Mussel Byssus‐Inspired MXene@Aramid Nanofibers Materials with Superior Endurance in Harsh Environments for Tunable EMI Shielding Performance,” *Adv. Mater. Interfaces*, vol. 9, no. 5, p. 2101359, Feb. 2022, doi: 10.1002/admi.202101359.

[12] B. Li *et al.*, “Bicontinuous, High-Strength, and Multifunctional Chemical-Cross-Linked MXene/Superaligned Carbon Nanotube Film,” *ACS Nano*, vol. 16, no. 11, pp. 19293–19304, Nov. 2022, doi: 10.1021/acsnano.2c08678.

[13] F. Shahzad *et al.*, “Electromagnetic interference shielding with 2D transition metal carbides (MXenes),” *Science*, vol. 353, no. 6304, pp. 1137–1140, Sep. 2016, doi: 10.1126/science.aag2421.

[14] Y. Zhang, “A flexible and strong reduced graphene oxide film for high-performance electromagnetic shielding,” *Compos. Commun.*, 2021.

[15] S. Lin, “Ultrathin nitrogen-doping graphene films for flexible and stretchable EMI shielding materials,” *J Mater Sci*, 2019.

[16] D. Lai, X. Chen, and Y. Wang, “Controllable fabrication of elastomeric and porous graphene films with superior foldable behavior and excellent electromagnetic interference shielding performance,” *Carbon*, vol. 158, pp. 728–737, Mar. 2020, doi: 10.1016/j.carbon.2019.11.047.

[17] B. Shen, W. Zhai, and W. Zheng, “Ultrathin Flexible Graphene Film: An Excellent Thermal Conducting Material with Efficient EMI Shielding,” *Adv. Funct. Mater.*, vol. 24, no. 28, pp. 4542–4548, Jul. 2014, doi: 10.1002/adfm.201400079.

[18] W.-L. Song *et al.*, “Magnetic and conductive graphene papers toward thin layers of effective electromagnetic shielding,” *J. Mater. Chem. A*, vol. 3, no. 5, pp. 2097–2107, 2015, doi: 10.1039/C4TA05939E.

[19] L. Zhang *et al.*, “Preparation and characterization of graphene paper for electromagnetic interference shielding,” *Carbon*, vol. 82, pp. 353–359, Feb. 2015, doi: 10.1016/j.carbon.2014.10.080.

[20] L. Paliotta *et al.*, “Highly conductive multilayer-graphene paper as a flexible lightweight electromagnetic shield,” *Carbon*, vol. 89, pp. 260–271, Aug. 2015, doi: 10.1016/j.carbon.2015.03.043.

[21] E. Zhou *et al.*, “Synergistic effect of graphene and carbon nanotube for high-performance electromagnetic interference shielding films,” *Carbon*, vol. 133, pp. 316–322, Jul. 2018, doi: 10.1016/j.carbon.2018.03.023.

[22] B. Zhao, C. Zhao, R. Li, S. M. Hamidinejad, and C. B. Park, “Flexible, Ultrathin, and High-Efficiency Electromagnetic Shielding Properties of Poly(Vinylidene Fluoride)/Carbon Composite Films,” *ACS Appl. Mater. Interfaces*, vol. 9, no. 24, pp. 20873–20884, Jun. 2017, doi: 10.1021/acsami.7b04935.

[23] Y. Yuan *et al.*, “Lightweight, flexible and strong core-shell non-woven fabrics covered by reduced graphene oxide for high-performance electromagnetic interference shielding,” *Carbon*, vol. 130, pp. 59–68, Apr. 2018, doi: 10.1016/j.carbon.2017.12.122.

[24] T. Wei, Z. Fan, G. Luo, C. Zheng, and D. Xie, “A rapid and efficient method to prepare exfoliated graphite by microwave irradiation,” *Carbon*, vol. 47, no. 1, pp. 337–339, Jan. 2009, doi: 10.1016/j.carbon.2008.10.013.

[25] “Electrical, mechanical, and thermal properties of exfoliated graphite/phenolic resin composite bipolar plate for polymer electrolyte membrane fuel cell - Sykam - 2015 - Polymer Engineering & Science - Wiley Online Library.” Accessed: Mar. 27, 2025. [Online]. Available: https://4spepublications.onlinelibrary.wiley.com/doi/abs/10.1002/pen.23959

[26] N. Sykam, N. D. Jayram, and G. Mohan Rao, “Exfoliation of graphite as flexible SERS substrate with high dye adsorption capacity for Rhodamine 6G,” *Appl. Surf. Sci.*, vol. 471, pp. 375–386, Mar. 2019, doi: 10.1016/j.apsusc.2018.11.082.

[27] Q. Wei *et al.*, “High-performance expanded graphite from flake graphite by microwave-assisted chemical intercalation process,” *J. Ind. Eng. Chem.*, vol. 122, pp. 562–572, Jun. 2023, doi: 10.1016/j.jiec.2023.03.020.

[28] N. Sykam, N. D. Jayram, and G. M. Rao, “Highly efficient removal of toxic organic dyes, chemical solvents and oils by mesoporous exfoliated graphite: Synthesis and mechanism,” *J. Water Process Eng.*, vol. 25, pp. 128–137, Oct. 2018, doi: 10.1016/j.jwpe.2018.05.013.

[29] C.-L. Ma, Z.-H. Hu, N.-J. Song, Y. Zhao, Y.-Z. Liu, and H.-Q. Wang, “Constructing mild expanded graphite microspheres by pressurized oxidation combined microwave treatment for enhanced lithium storage,” *Rare Met.*, vol. 40, no. 4, pp. 837–847, Apr. 2021, doi: 10.1007/s12598-020-01625-9.

[30] “Expanded Graphite Modified by CTAB-KBr/H3PO4 for Highly Efficient Adsorption of Dyes | Journal of Polymers and the Environment.” Accessed: Mar. 27, 2025. [Online]. Available: https://link.springer.com/article/10.1007/s10924-017-1019-0?utm_source=getftr&utm_medium=getftr&utm_campaign=getftr_pilot&getft_integrator=sciencedirect_contenthosting

[31] X.-J. Yu, J. Wu, Q. Zhao, and X.-W. Cheng, “Preparation and characterization of sulfur-free exfoliated graphite with large exfoliated volume,” *Mater. Lett.*, vol. 73, pp. 11–13, Apr. 2012, doi: 10.1016/j.matlet.2011.11.078.

[32] J. Yan, Z. Fan, T. Wei, W. Qian, M. Zhang, and F. Wei, “Preparation of exfoliated graphite containing manganese oxides with high electrochemical capacitance by microwave irradiation,” *Carbon*, vol. 47, no. 14, pp. 3371–3374, Nov. 2009, doi: 10.1016/j.carbon.2009.08.001.

[33] Y.-L. Chen, C.-H. Hsiao, J.-Y. Ya, and P.-Y. Hsieh, “Preparation of expanded graphite with (NH4)2S2O8 and H2SO4 by using microwave irradiation,” *J. Taiwan Inst. Chem. Eng.*, vol. 154, p. 105026, Jan. 2024, doi: 10.1016/j.jtice.2023.105026.

[34] N. Sykam and K. K. Kar, “Rapid synthesis of exfoliated graphite by microwave irradiation and oil sorption studies,” *Mater. Lett.*, vol. 117, pp. 150–152, Feb. 2014, doi: 10.1016/j.matlet.2013.12.003.

[35] J. He, M. Yuan, H. Ren, T. Song, and Y. Zhang, “The electrochemical preparation and characterization of sulfur-free expanded graphite,” *J. Chem. Sci.*, vol. 135, no. 1, p. 17, Feb. 2023, doi: 10.1007/s12039-023-02138-5.

[36] X. H. Wei, L. Liu, J. X. Zhang, J. L. Shi, and Q. G. Guo, “HClO4–graphite intercalation compound and its thermally exfoliated graphite,” *Mater. Lett.*, vol. 63, no. 18, pp. 1618–1620, Jul. 2009, doi: 10.1016/j.matlet.2009.04.030.

[37] T. Liu, R. Zhang, X. Zhang, K. Liu, Y. Liu, and P. Yan, “One-step room-temperature preparation of expanded graphite,” *Carbon*, vol. 119, pp. 544–547, Aug. 2017, doi: 10.1016/j.carbon.2017.04.076.

[38] X. Lv, X. Wang, Z. Huang, X. Liu, and C. Lv, “Preparation of exfoliated graphite intercalated with nitrogen dioxide by direct gas-phase processing,” *Mater. Lett.*, vol. 136, pp. 48–51, Dec. 2014, doi: 10.1016/j.matlet.2014.08.009.

[39] C. Dai *et al.*, “Preparation of low-temperature expandable graphite as a novel steam plugging agent in heavy oil reservoirs,” *J. Mol. Liq.*, vol. 293, p. 111535, Nov. 2019, doi: 10.1016/j.molliq.2019.111535.

[40] T. Peng, B. Liu, X. Gao, L. Luo, and H. Sun, “Preparation, quantitative surface analysis, intercalation characteristics and industrial implications of low temperature expandable graphite,” *Appl. Surf. Sci.*, vol. 444, pp. 800–810, Jun. 2018, doi: 10.1016/j.apsusc.2018.03.089.

[41] Y. V. Berestneva, E. V. Raksha, A. A. Voitash, G. M. Arzumanyan, and M. V. Savoskin, “Thermally expanded graphite from graphite nitrate cointercalated with ethyl formate and acetic acid: morphology and physicochemical properties,” *J. Phys. Conf. Ser.*, vol. 1658, no. 1, p. 012004, Oct. 2020, doi: 10.1088/1742-6596/1658/1/012004.

[42] J. Li *et al.*, “Beneficiation of ultra-large flake graphite and the preparation of flexible graphite sheets from it,” *New Carbon Mater.*, vol. 34, no. 2, pp. 205–210, Apr. 2019, doi: 10.1016/S1872-5805(19)60012-0.

[43] M.-Y. Shen *et al.*, “Preparation of expandable graphite and its flame retardant properties in HDPE composites,” *Polym. Compos.*, vol. 38, no. 11, pp. 2378–2386, 2017, doi: 10.1002/pc.23820.

[44] M. M. Ardestani, S. Mahpishanian, B. F. Rad, M. Janmohammadi, and M. Baghdadi, “Preparation and characterization of room-temperature chemically expanded graphite: Application for cationic dye removal,” *Korean J. Chem. Eng.*, vol. 39, no. 6, pp. 1496–1506, Jun. 2022, doi: 10.1007/s11814-022-1084-5.

[45] Y. F. Yang, X. J. Zhang, and X. Xu, “Preparation and Characteristics of Expanded Graphite,” *Adv. Mater. Res.*, vol. 189–193, pp. 2695–2698, 2011, doi: 10.4028/www.scientific.net/AMR.189-193.2695.

[46] M. Elbidi, M. F. M. G. Resul, S. A. Rashid, and M. A. M. Salleh, “Preparation of eco-friendly mesoporous expanded graphite for oil sorption,” *J. Porous Mater.*, vol. 30, no. 4, pp. 1359–1368, Aug. 2023, doi: 10.1007/s10934-023-01428-0.

[47] J. Li, Q. Liu, and H. Da, “Preparation of sulfur-free exfoliated graphite at a low exfoliation temperature,” *Mater. Lett.*, vol. 61, no. 8, pp. 1832–1834, Apr. 2007, doi: 10.1016/j.matlet.2006.07.142.

[48] J. He, L. Song, H. Yang, X. Ren, and L. Xing, “Preparation of Sulfur-Free Exfoliated Graphite by a Two-Step Intercalation Process and Its Application for Adsorption of Oils,” *J. Chem.*, vol. 2017, no. 1, p. 5824976, 2017, doi: 10.1155/2017/5824976.

[49] B. Hou, H. Sun, T. Peng, X. Zhang, and Y. Ren, “Rapid preparation of expanded graphite at low temperature,” *New Carbon Mater.*, vol. 35, no. 3, pp. 262–268, Jun. 2020, doi: 10.1016/S1872-5805(20)60488-7.

[50] X. Tang *et al.*, “Study on the mechanism of expanded graphite to improve the fading resistance of the non-asbestos organic composite braking materials,” *Tribol. Int.*, vol. 180, p. 108278, Feb. 2023, doi: 10.1016/j.triboint.2023.108278.

[51] X. H. Wei, L. Liu, J. X. Zhang, J. L. Shi, and Q. G. Guo, “The preparation and morphology characteristics of exfoliated graphite derived from HClO4–graphite intercalation compounds,” *Mater. Lett.*, vol. 64, no. 9, pp. 1007–1009, May 2010, doi: 10.1016/j.matlet.2009.11.025.

[52] G. Carotenuto *et al.*, “Laser-Induced Thermal Expansion of H2SO4-Intercalated Graphite Lattice,” ACS Publications. Accessed: Mar. 27, 2025. [Online]. Available: https://pubs.acs.org/doi/abs/10.1021/jp512800j

[53] R. Trusovas, K. Ratautas, G. Račiukaitis, and G. Niaura, “Graphene layer formation in pinewood by nanosecond and picosecond laser irradiation,” *Appl. Surf. Sci.*, vol. 471, pp. 154–161, Mar. 2019, doi: 10.1016/j.apsusc.2018.12.005.

[54] “Laser‐Assisted Ultrafast Exfoliation of Black Phosphorus in Liquid with Tunable Thickness for Li‐Ion Batteries - Zheng - 2020 - Advanced Energy Materials - Wiley Online Library.” Accessed: Mar. 27, 2025. [Online]. Available: https://advanced.onlinelibrary.wiley.com/doi/abs/10.1002/aenm.201903490
